# Cholinergic modulation of sensory processing in awake mouse cortex

**DOI:** 10.1038/s41598-021-96696-8

**Published:** 2021-09-01

**Authors:** Javier Jimenez-Martin, Daniil Potapov, Kay Potapov, Thomas Knöpfel, Ruth M. Empson

**Affiliations:** 1 Department of Physiology, Biomedical Sciences, Brain Health Research Centre and Brain Research NZ, University of Otago, Dunedin, New Zealand; 2 Faculty of Medicine, Department of Brain Sciences, Imperial College London, London, UK

**Keywords:** Neuroscience, Neural circuits, Sensorimotor processing, Sensory processing, Somatosensory system, Synaptic transmission, Neurological disorders

## Abstract

Cholinergic modulation of brain activity is fundamental for awareness and conscious sensorimotor behaviours, but deciphering the timing and significance of acetylcholine actions for these behaviours is challenging. The widespread nature of cholinergic projections to the cortex means that new insights require access to specific neuronal populations, and on a time-scale that matches behaviourally relevant cholinergic actions. Here, we use fast, voltage imaging of L2/3 cortical pyramidal neurons exclusively expressing the genetically-encoded voltage indicator Butterfly 1.2, in awake, head-fixed mice, receiving sensory stimulation, whilst manipulating the cholinergic system. Altering muscarinic acetylcholine function re-shaped sensory-evoked fast depolarisation and subsequent slow hyperpolarisation of L2/3 pyramidal neurons. A consequence of this re-shaping was disrupted adaptation of the sensory-evoked responses, suggesting a critical role for acetylcholine during sensory discrimination behaviour. Our findings provide new insights into how the cortex processes sensory information and how loss of acetylcholine, for example in Alzheimer’s Disease, disrupts sensory behaviours.

## Introduction

Acetylcholine (ACh) is a widespread neurotransmitter and neuromodulator long known to act throughout the central nervous system across a variety of circuitries and timescales^1–7^. Cholinergic neurons of the basal forebrain (BF) form organised and widespread projections to release ACh across the whole cortex^8^. These extensive cholinergic projections are critical for many behaviours such as body awareness, attention, sleep and arousal and also for motor skill development, learning, memory and cognition, in both health and disease^9–18^.

These multiple roles of ACh, for such a variety of behaviours, occurring across various cortical areas, make it particularly challenging to understand how this critical neuromodulator acts. Specific optical stimulation and electrophysiology in vitro have significantly advanced our understanding of how nicotinic and muscarinic cholinergic actions alter single pyramidal neuron, inhibitory interneuron and cortical network activity^19–25^*.* A renaissance of EEG, and whole-cell electrophysiology, with optogenetic stimulation also show how basal forebrain cholinergic activity in vivo promotes attention, wakefulness and visual perception by influencing cortical neuron gain control, signal-to-noise and synchrony^26–33^.

Here, we focus on the somatosensory cortex, in particular layer 2/3 (L2/3) pyramidal neuron activity, since L2/3 amplifies layer 5 (L5) pyramidal neuron somatosensory output^34^ and provides a long-range broadcasting network connecting different cortical regions^35–38^. Somatosensory L2/3 cortical pyramidal neuron activity is powerfully influenced by ACh^19,24,25,33^ and BF projections to this cortical layer are particularly dense^8^. The majority of L2/3 pyramidal neurons fire sparsely as a consequence of precisely balanced recurrent excitation and feed-forward and feedback inhibition^39–41^ and ACh modifies this balance to influence L2/3 pyramidal neuron activity^20,21^.

Despite this knowledge, how ACh modifies L2/3 cortical pyramidal neuron activity during awake sensory processing remains unanswered. Perhaps part of the challenge has been our inability to access widespread but specific cortical circuit activity on a time scale that matches ACh actions, and during behaviourally relevant responses in the awake animal. To address those experimental issues, we established fast voltage imaging of L2/3 cortical-wide activity in awake, head-fixed mice, asking specifically how ACh influences real-time cortical activity evoked by simple sensory stimulation. We took advantage of transgenic mice expressing the genetically-encoded voltage indicator (GEVI), Butterfly 1.2, selectively in L2/3 pyramidal neurons^42–45^. In quiet, head-fixed mice, different somatosensory tactile stimuli evoked distinct spatial and temporal patterns of L2/3 pyramidal neuron depolarisation and hyperpolarisation, and manipulating ACh function significantly re-shaped these patterns. Conceptually, our results extend recent, elegant work in vitro*,* showing how cholinergic inhibition and disinhibition operate together within the canonical cortical circuitry to sustain awake sensory processing. More widely, ACh influenced the response to a second, closely-timed sensory stimulation, known as sensory adaptation, with implications for maintaining sensory awareness and accurate sensory discrimination behaviours.

## Results

### Distinct sensory-evoked L2/3 pyramidal neuron membrane voltage patterns evoked by brief whisker and forepaw stimulation

**Figure 1:**
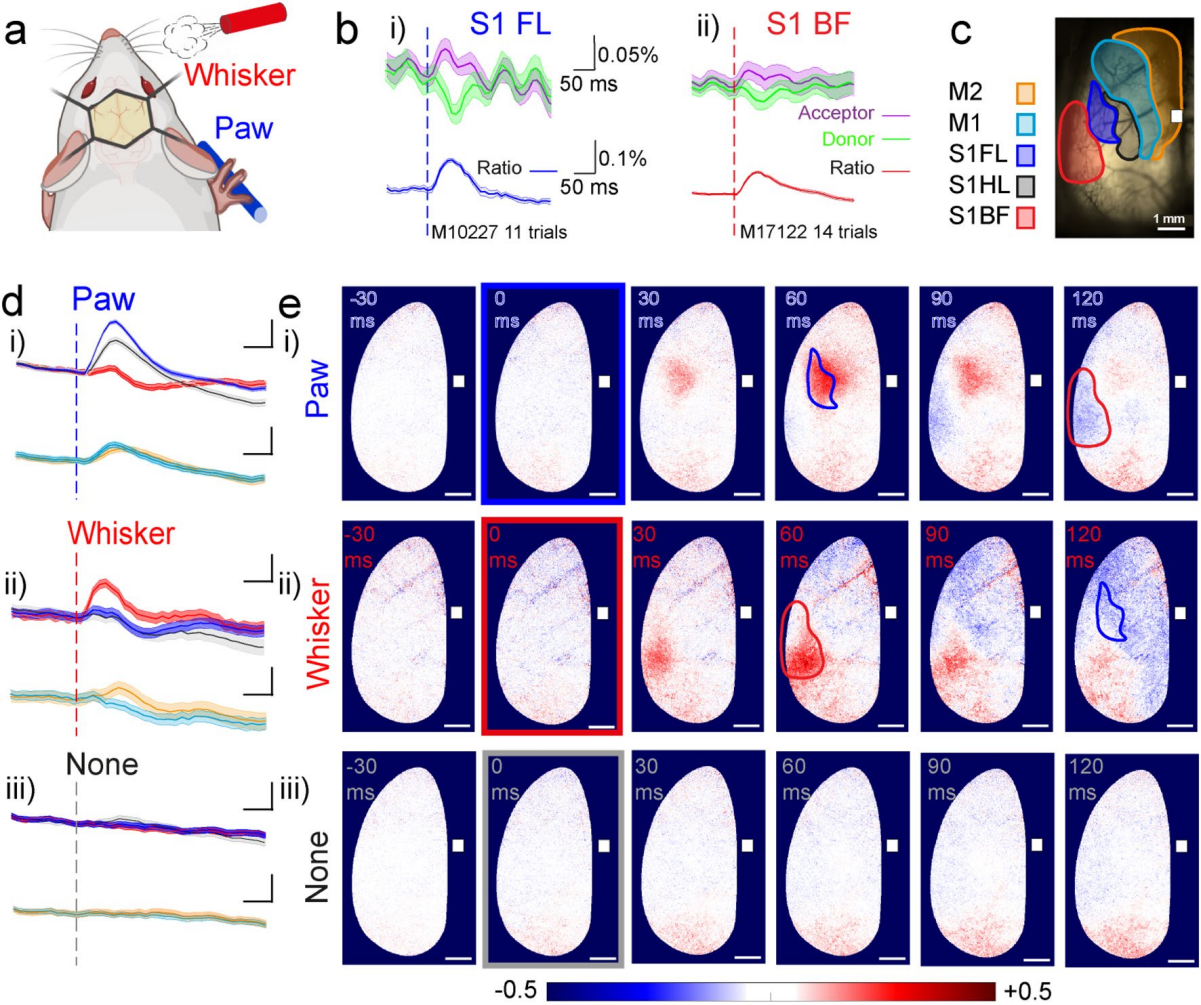
Distinct voltage response patterns across cortical areas following forepaw and whisker sensory stimulation (“Results”; “Discussion”). (**a**) Diagram showing awake, head-fixed mice expressing VSFP Butterfly 1.2 in Layer 2/3 cortical pyramidal neurons, with a thinned-skull cranial window receiving forepaw and whisker stimulation. Blue and red colours indicate paw and whisker stimulation, respectively in this and all following figures. Mouse image created with Biorender.com. (**b**) (i) Sensory-evoked donor (green) and acceptor (purple) fluorescence responses (ΔF/F) induced by light forepaw stimulation spatially averaged over the contralateral forelimb area (S1FL) in a single mouse id M10227. Corresponding ΔR/R ratiometric traces represent the voltage response, blue trace. Vertical dashed line indicates stimulation onset. n = 11 trials. (ii) Same as in (i), sensory-evoked responses to a short air puff directed to the whiskers, onset indicated by the vertical red dashed line. Responses are spatially averaged over the contralateral Barrel Field (S1BF) for single mouse id M17122, n = 14 trials. (**c**) Through-skull cranial window with mapped areas according to “The Mouse Brain in Stereotaxic Coordinates”^48^, relative to bregma. (M1, Primary motor cortex; M2, secondary motor cortex; S1HL, Hindlimb area of the primary sensory cortex; S1FL, Forelimb area of the primary sensory cortex; S1BF, Barrel field of the primary sensory cortex). Bregma shown with a white square. (**d**) Sensory-evoked voltage responses spatially averaged from areas S1FL (blue), S1HL (black), SIBF (red), M1 (cyan) and M2 (orange) in response to forepaw stimulation (i), whisker stimulation (ii) and control trials with no stimulation (iii). Note the hyperpolarisation of S1BF in response to paw stimulation, (i), and the hyperpolarisation of S1FL in response to whisker stimulation (ii). Vertical dashed line indicates stimulation onset. ΔR/R for ratiometric trace. (Paw stimulation n = 118 trials, 12 mice. Whisker stimulation n = 52 trials, 10 mice. No stimulation n = 152 trials, 12 mice), scalebar 0.1% ΔR/R and 50 ms. (**e**) Sensory-evoked voltage maps in response to paw stimulation (i), whiskers stimulation (ii) and no stimulation trials, none (iii) at 30 ms before (− 30 ms), at the stimulation time (0 ms, framed in blue, paw, or red, whisker) and selected times after each stimulation. Maps contain the same data as the traces in D. Depolarised pixels red, + 0.5% ΔR/R and hyperpolarised pixels blue, − 0.5% ΔR/R. Scale bar 1 mm. Bregma shown with a white square. Paw stimulation evoked a depolarization in S1FL (area outlined in blue) 60 ms after the stimulation and a hyperpolarization of S1BF (area outlined in red) 120 ms after the stimulation. Whisker stimulation evoked a depolarization in S1BF 60 ms after stimulation and a hyperpolarization, in S1FL 120 ms after the stimulation. Side by side videos of whisker and paw stimulation are found in Supplementary Video 1.

We imaged population voltage transients of L2/3 pyramidal neurons across the hemisphere contralateral to forepaw and whisker stimulation (Fig. 1a). The GEVI Butterfly 1.2 reports membrane voltage by anticorrelated changes in fluorescence intensity emission from the FRET donor (mCitrine) and acceptor (mKate2) fluorescent proteins^44^. The ratiometric signal (Fig. 1b i), blue, for paw and ii) red, for whisker traces) reflects depolarisation (decrease in donor and increase in acceptor fluorescence intensities) and hyperpolarisation (increase in donor and decrease in acceptor) of the membrane voltage. These responses uniquely report membrane voltage of all, and only, L2/3 pyramidal neurons^42^, and are strongly biased by voltage transients in their apical dendrites^46^. We calculated the ratio of the donor and acceptor fluorescence changes on a pixel-by-pixel basis using previously developed methodologies^47^ within cortical areas delineated by Paxinos coordinates (Fig. 1b,c)^48^.

We delivered one sensory stimulus during each recording trial and then classified each trial as “movement” when the mouse actively or spontaneously moved, or “quiet” when we only detected passive respiratory movements of the mouse. All our analysis used “quiet” trials, to avoid any technical complications from movement-related optical signals. Supplementary Fig. 3 shows all trials from Whisker and Paw responses from all mice and removal of trials where the mouse moved (see Methods and Supplementary Figs. 1, 2) or where the peak amplitude was < 2SD of the baseline signal.

A brief (2 ms duration), light vibration of the forepaw depolarises L2/3 pyramidal neurons in the forelimb area of S1, S1FL, whereas an air puff (10 psi, 20 ms duration) directed to the whiskers depolarises the barrel field area, S1BF. In the early part of the response, immediately after the stimulus, the anticorrelated increased acceptor and decreased donor fluorescence (Fig. 1b i, ii) indicating membrane depolarisation of L2/3 pyramidal neurons^44^. Note that in response to forepaw stimulation a visible, smaller increase of the donor and decrease of the acceptor fluorescence in S1FL begins around 100 ms after the stimulus (Fig. 1b i), indicating a slow membrane hyperpolarisation. This slow part of the response is consistent with activation of feed-forward surround inhibition of L2/3 pyramidal neurons^49^ and the broad spatial influence of long-lasting inhibition seen in awake mice after visual stimulation^50^.

In response to forepaw or whisker stimulation a 10 ms delay preceded rapid depolarisations in the S1FL and S1BF areas. The peak depolarising response in the S1FL to forepaw stimulation was slower and larger than the response in the S1BF after whisker stimulation (amplitude following forepaw stimulation in S1FL was 0.195 ± 0.007% compared with 0.156 ± 0.007% in S1BF following whisker stimulation (Mann–Whitney test, U = 2049, *p* = 0.0005) at 59.83 ± 1.13 ms (peak of forepaw-evoked responses) and 50.58 ± 1.95 ms (peak of whiskers-evoked responses) after stimulation respectively (Mann–Whitney test, U = 1853, *p* < 0.0001), paw stimulation n = 118 trials, 12 mice and whisker stimulation n = 52 trials, 10 mice; see also Supplementary Table 1.

These Butterfly 1.2 reported times to peak and decay of the depolarisations are within the temporal range of previous in vivo sensory-evoked cortical responses from L2/3 pyramidal neurons obtained using patch-clamp electrophysiology, synthetic voltage-sensitive dyes and the chimeric Butterfly GEVI^35,47,51–54^. Our recordings largely reflect sub-threshold, not action potential activity^42^, with sufficient signal-to-noise to report single trial responses (Supplementary Fig. 1). Their time-scale accords with the sub-threshold components of voltage responses obtained in vivo with fast, high-gain GEVIs that also report high frequency somatic action potentials^55,56^.

It is important to note that the responses in Fig. 1 and all subsequent main figures are from quiet trials and we considered them to be predominantly sensory responses. In contrast, when spontaneous mouse movement occurred within 100 ms of sensory stimulation (Supplementary Fig. 2) we observed slower more widespread optical signals of all cortical areas, time-locked with the mouse movement and distinct from the initial fast depolarising sensory-evoked response.

### Distinct propagation patterns of sensory-evoked L2/3 pyramidal neuron membrane voltage

We spatially averaged voltage signals for anatomically registered areas (Fig. 1c) to generate time traces of L2/3 voltage activity (Fig. 1d) shown alongside spatially averaged (by pixel) maps of L2/3 voltage activity (Fig. 1e). Together the maps and traces show how forepaw stimulation evoked widespread and rapid depolarisation of the primary sensory cortical area, S1FL, and neighbouring cortical areas including primary motor cortex (M1) and secondary motor cortex (M2) with noticeably rapid depolarisation of distant, rostral M2^35^. The time-scale of these depolarisations was consistent with activation of direct excitatory connections between S1FL and M2^54^, for more detail on timings see Supplementary Table 1. Markedly, forepaw stimulation noticeably hyperpolarised the barrel field, S1BF (Fig. 1d i, red trace), and whisker stimulation hyperpolarised the forelimb areas S1FL (Fig. 1d ii, blue trace).

Whisker stimulation evoked a fast depolarisation of S1BF largely contained within this area, with a slow, small depolarisation of M2 in the vibrissae motor area^36^, visibly distinct from forepaw activation of M2, but with a similar time to peak (Fig. 1e, and Supplementary Table 1). Notably, the limb areas, S1FL, hind limb area of the primary sensory cortex, S1HL, and forebrain area M1 also slowly hyperpolarised in response to whisker stimulation, perhaps signifying reduced attention^57^. Side-by-side videos of whisker and paw stimulation can be found in Supplementary Video 1. Controls confirmed the absence of responses in sensory or motor areas from the same animals recorded under identical conditions when quiet, but without sensory stimulation (Fig. 1d iii, e iii and Supplementary Fig. 3).

### Sensory-evoked hyperpolarisation of L2/3 pyramidal neurons provides functional cross-modal inhibition

**Figure 2:**
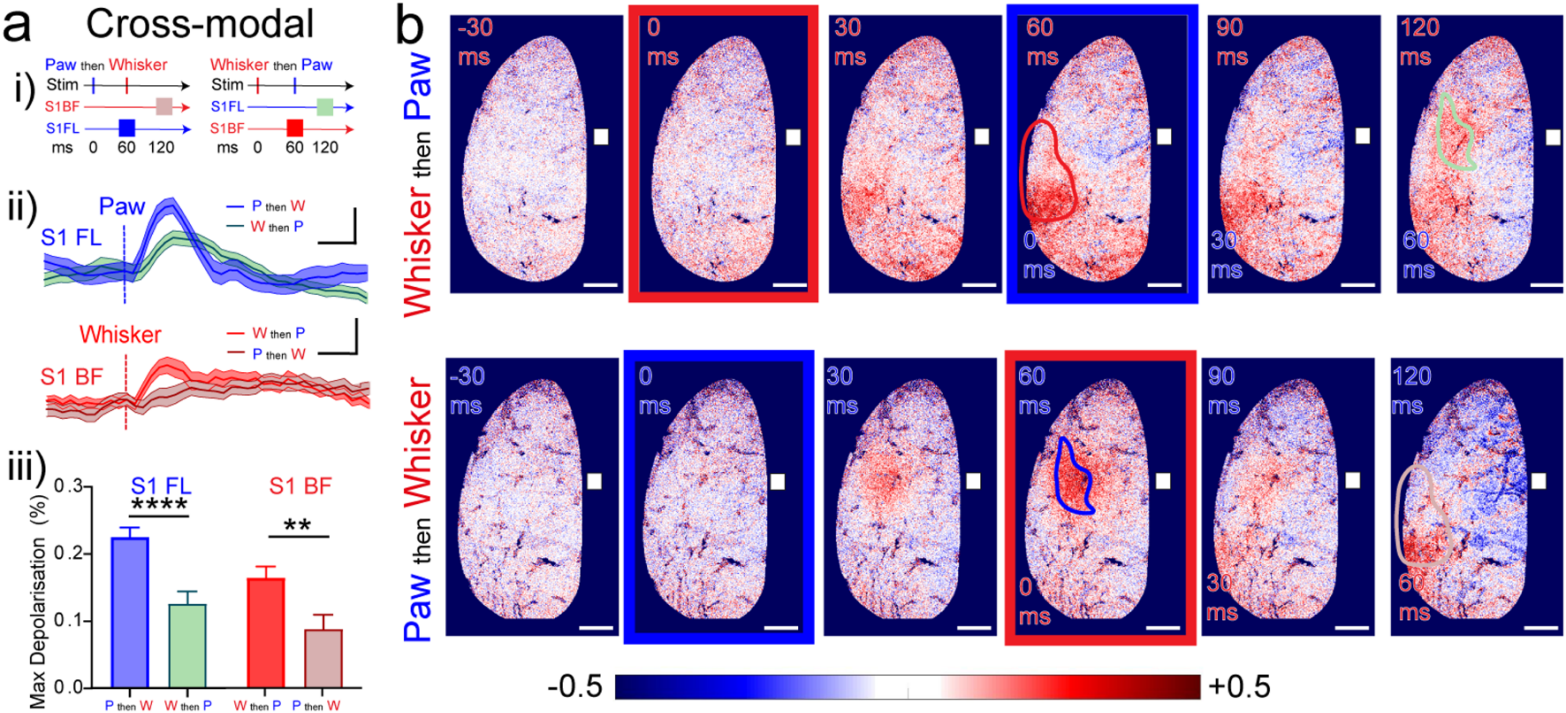
Cross Modal Inhibition evoked by Whisker and Paw Stimulation (“Results”; “Discussion”). (**a**) Cross-modal sensory stimulation. (i) Timeline of the cross-modal sensory stimulation. In “paw then whisker (P then W)” experiments the forepaw was stimulated first and then 60 ms later an air puff was delivered to the mouse whiskers. Blue bar represents the time of the peak S1FL response, 60 ms after stimulation. Pink bar represents the time of the peak S1BF response 60 ms after stimulation, ie 120 ms. In “Whisker then Paw (W then P)” experiments the whisker was stimulated first and 60 ms later a light stimulation was delivered to the forepaw. Red bar represents the time of the peak S1BF response, 60 ms after stimulation. Green bar represents the time of the peak S1FL response 60 ms after stimulation, ie 120 ms. (ii) Sensory-evoked voltage responses spatially averaged across S1FL after Paw stimulation, of “P then W” (blue trace) and of “W then P” (pale green trace), note the reduced amplitude of the paw-evoked response when stimulated after the whiskers (pale green trace). Sensory-evoked voltage responses spatially averaged across S1BF after Whisker stimulation, of “W then P” (red trace) and of “P then W” (pale red trace). Note the reduced amplitude of the whisker-evoked response when stimulated after the paw (pale red trace). Scalebar 0.1% ΔR/R and 50 ms. (iii) Maximum depolarization amplitude in S1FL from “P then W” and “W then P” trials and in the S1BF in “W then P” and “P then W” trials. (S1FL Mann–Whitney test, U = 152, *p* < 0.0001; S1BF Mann–Whitney test, U = 191, *p* = 0.0014). **p < 0.01, ****p < 0.0001. (Paw then whisker n = 26 trials, 4 mice. Whisker then Paw n = 29 trials, 4 mice). (**b**) Voltage maps from paw then whisker stimulation (top) and from whisker then paw stimulation experiments. Maps contain the same data as the traces in F ii) and are framed with colours indicating times of stimulation, blue, paw, red, whisker. Depolarised pixels red, + 0.5% ΔR/R and hyperpolarised pixels blue, − 0.5% ΔR/R. Scalebar 1 mm. Bregma shown with a white square. Data are mean ± SEM. Scale bars 0.1% ΔR/R and 100 ms. Supplementary Fig. 3 shows the depolarisation/hyperpolarisation red/blue colour-coded maps for all individual mice.

The spatially segregated sensory-evoked hyperpolarisations, for example in S1BF after forepaw stimulation, indicate the recruitment of L2/3 cortical inhibitory circuits. To test for functional inhibition, we first stimulated the forepaw and then 60 ms later (at the peak of the S1FL response) we stimulated the whiskers in the same mouse (Fig. 2a i). As predicted, prior forepaw stimulation functionally inhibited S1BF, significantly reducing the amplitude of the response to whisker stimulation by around 50%. Similarly, prior whisker stimulation significantly reduced the amplitude of the forepaw-evoked response in S1FL (Fig. 2a ii,iii). The reduced depolarisation of the whisker and forepaw areas, 60 ms after the second stimulation, is also evident in Fig. 2b. These results exemplify precisely timed, long-range and reciprocal inhibition of the L2/3 pyramidal neuron broadcast network for cross-modal sensory integration. Our findings may illustrate effective silencing of non-salient sensory cortical areas, for example silencing whisker areas after forepaw stimulation, because forepaw perception may be more relevant at that time.

### Scopolamine, a muscarinic cholinergic antagonist re-shapes forepaw-evoked L2/3 pyramidal neuron membrane voltage patterns

**Figure 3:**
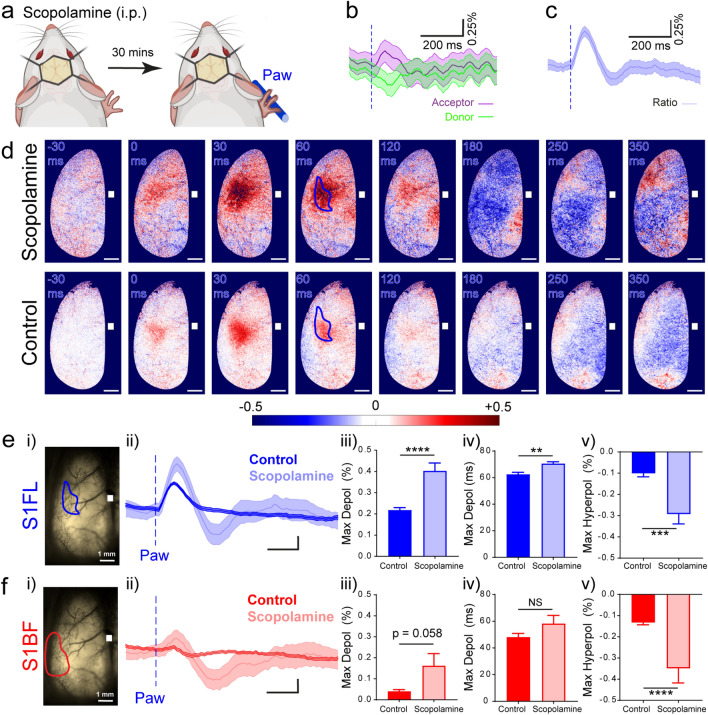
Systemic scopolamine, a cholinergic antagonist, re-shapes the amplitude and timing of tactile-evoked voltage responses in cortical sensory areas (“Results”; “Discussion”). (**a**) Schematic methods. Mice received a single intraperitoneal (i.p.) injection of 1 mg/kg of scopolamine, 30 min later mice were head-fixed and imaged during paw stimulation. Mouse images created with Biorender.com. (**b**) Average sensory-evoked donor (green) and acceptor (purple) fluorescence responses (ΔF/F) induced by paw stimulation spatially averaged over the contralateral forelimb area (S1FL) in scopolamine-treated mice. Vertical dashed line indicates stimulation onset. (n = 17 trials, 6 mice). (**c**) Average sensory-evoked voltage responses, ratio ΔR/R, from the same trials as in B. Vertical dashed line indicates stimulation onset. (**d**) Sensory-evoked voltage maps in response to paw stimulation 30 ms before (− 30 ms), at the stimulation time (0 ms) and selected times after stimulation in control and scopolamine-treated mice. Maps contain the same data as the traces in E and F. Depolarised pixels red, + 0.5% ΔR/R and hyperpolarised pixels blue, − 0.5% ΔR/R. Scale bar 1 mm. Bregma is a white square. (Scopolamine n = 17 trials, 6 mice. Control n = 58 trials, 6 mice). Side by side videos of responses to paw stimulation in mice before and after scopolamine-treatment can be found in Supplementary Video 2. (**e**) (i) Through-skull cranial window with mapped S1FL according to “The Mouse Brain in Stereotaxic Coordinates”^48^, relative to bregma, shown with a white square. (ii) Average voltage traces from the S1FL area in response to paw stimulation in control and scopolamine-treated mice. Vertical dashed line represents stimulus onset. Scalebar 0.1% ΔR/R and 100 ms. (iii) Maximum depolarisation amplitude, Mann–Whitney test, U = 115, *p* < 0.0001. (iv) Time of maximum depolarization, Mann–Whitney test, U = 252, *p* = 0.0010. (v) Maximum hyperpolarisation amplitude, Mann–Whitney test, U = 199, *p* = 0.0001. (**f**) (i) Barrel field of the primary sensory cortex (S1BF) mapped as in E. (ii) Average voltage traces from the S1BF area in response to Paw stimulation in control and scopolamine-treated mice. Vertical dashed line indicates stimulation onset. Scalebar 0.1% ΔR/R and 100 ms. (iii) Maximum depolarisation amplitude, Mann–Whitney test, U = 318, *p* = 0.058. (iv) Time of maximum depolarization, Mann–Whitney test, U = 368.5, *p* = 0.111. v) Maximum hyperpolarisation amplitude, Mann–Whitney test, U = 126, *p* < 0.0001. Data are mean ± SEM; ***p < 0.001, NS non-significant.

Next, we sought to test how manipulating cholinergic actions influenced the timing and behaviour of the L2/3 network, by recording responses to forepaw stimulation after a systemic injection of scopolamine, a muscarinic cholinergic antagonist (Fig. 3). Under these conditions, forepaw stimulation evoked a fast depolarisation followed by a large slow hyperpolarisation peaking at around 150–180 ms in S1FL (Fig. 3b,c) that spread very widely across the cortex rostrally and caudally (Fig. 3d, Supplementary Fig. 4). Both fast depolarisation and slower hyperpolarisation were significantly larger than in control, with significantly increased time to peak of the initial depolarisation. We also observed a weaker, slower depolarisation of S1FL at 200–300 ms after stimulation in scopolamine (Fig. 3e ii). A possible explanation is that loss of a slow muscarinic-dependent silencing of layer 4 (L4) pyramidal neurons^19^ permits a slow L4 to L2/3 pyramidal neuron activation.

In Fig. 3f i, in the presence of scopolamine, the normal forepaw-evoked hyperpolarisation of S1BF became a fast depolarisation (Fig. 2f iii) followed by a much longer, larger hyperpolarisation (Fig. 2f v). Side-by-side videos of responses to paw stimulation in mice before and after scopolamine-treatment are to be found in Supplementary Video 2.

As seen in Fig. 3d, forepaw stimulation evoked widespread depolarisation of the frontal areas in mice treated with scopolamine; the amplitude of the depolarisation of M1 increased three-fold in the presence of scopolamine, from 0.112 ± 0.0008% in controls to 0.345 ± 0.008% (Mann–Whitney test, U = 163, *p* = 0.0005), with the time to peak increasing from 61.96 ± 2.29 ms to 76.67 ± 3.61 ms (Mann–Whitney test, U = 184.5, *p* = 0.001). Similar to S1FL, the depolarisation of M1 was followed by an extensive slow hyperpolarisation, followed by a much later depolarisation.

### Depletion of cholinergic fibres in the cortex re-shapes forepaw sensory-evoked L2/3 pyramidal neuron membrane voltage patterns in a similar manner to scopolamine

**Figure 4:**
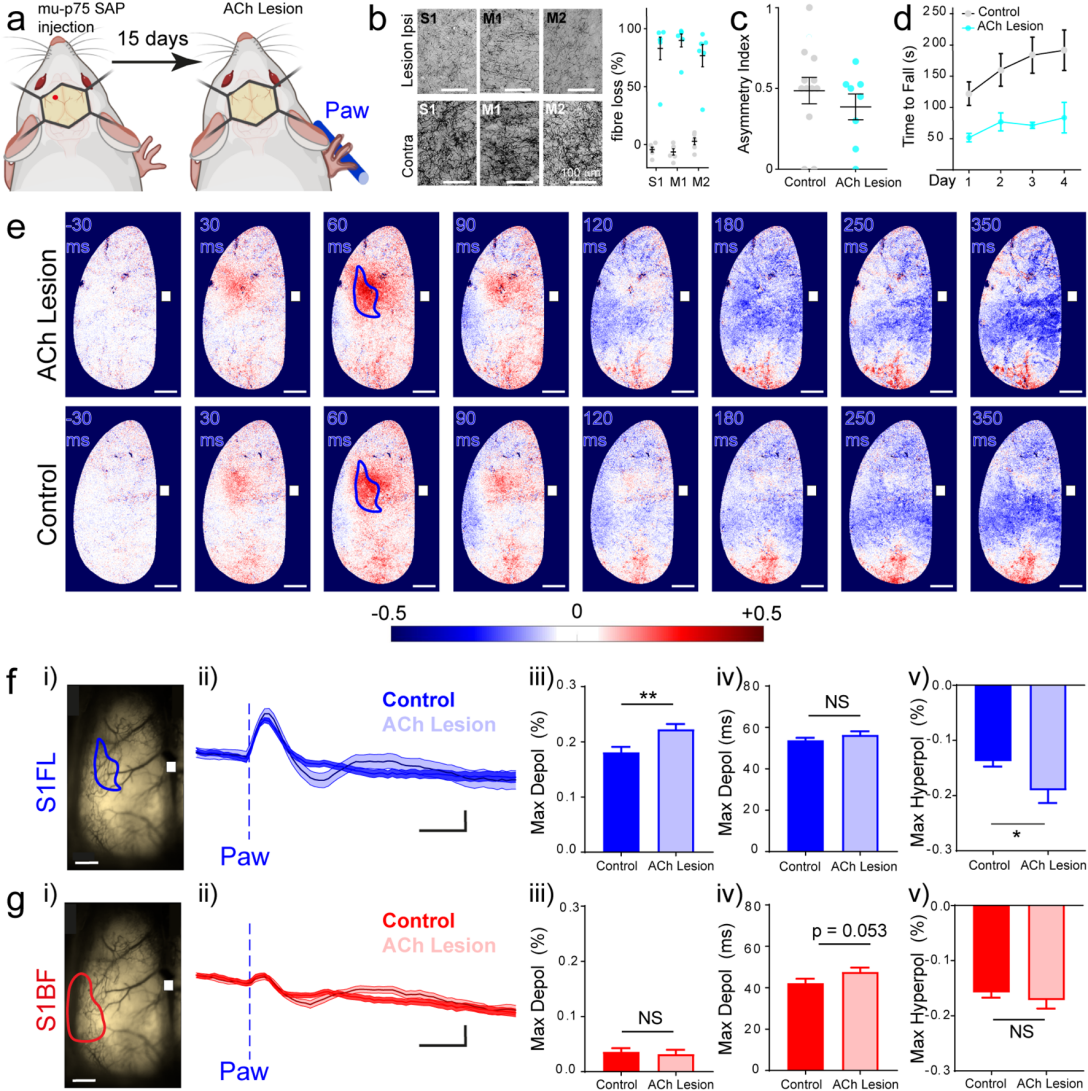
Specific lesion of cortical cholinergic fibres re-shapes the amplitude and timing of tactile-evoked voltage responses in sensory cortical areas. (“Results”; “Discussion”). (**a**) Schematic methods. Mice received a unilateral cortical injection of mu-p75 SAP or rabbit IgG SAP Control into the left hemisphere, red dot indicates injection site. 15 days later mice were head-fixed and imaged during paw stimulation. Mouse images created with Biorender.com. (**b**) Reduction of cholinergic fibres in the contralateral cortex (visualised with acetylcholinesterase staining 15 days after mu-p75SAP injection). Percentage of cholinergic fibre loss relative to the contralateral (non-injected hemisphere). Two-way ANOVA, Areas F (2, 30) = 0.08495, *p* = 0.9188, Groups F (1, 30) = 276.4, *p* < 0.0001; Interaction F (2, 30) = 1.586, *p* = 0.2214. Data are mean ± SEM. (Cholinergic (ACh) lesion n = 6 mice, control n = 6 mice). (**c**) The forelimb asymmetry index is unaffected 15 days after ACh lesion. Wilcoxon Signed Rank Test, compared to 0.5, theoretical value of symmetrical use of the forelimbs, *p* ≥ 0.05 ACh lesion *p* = 0.2812, Control *p* = 0.9297; and unpaired t-test, t _(18)_ = 0.8387, *p* = 0.4126 (ACh lesion n = 12, control n = 8). (**d**) ACh-lesioned mice show impaired performance on the accelerating rotarod 15 days after the lesion, compared with time-matched Control mice (Two-way repeated measurements ANOVA, Interaction F (3, 27) = 1.484, *p* = 0.2411; time F (3, 27) = 7.071, *p* = 0.0086; groups F (1, 9) = 6.993, *p* = 0.0267 (ACh lesion n = 4, control n = 7). (**e**) Sensory-evoked voltage maps in response to paw stimulation at selected times before and after stimulation (0 ms) in ACh-lesioned and Control mice. Scale bar is 1 mm. Bregma shown with a white square. Depolarised pixels red, + 0.5% ΔR/R and hyperpolarised pixels blue, − 0.5% ΔR/R. Maps contain the same data as the traces in F and G. (ACh lesion n = 58 trials, 6 mice. Control n = 61 trials, 6 mice, all 15 days after injection). Side by side videos of responses to forepaw stimulation in mice 15 days after mu-p75SAP injection and control can be found in Supplementary Video 3. (**f**) (i) Through-skull cranial window with mapped areas according to “The Mouse Brain in Stereotaxic Coordinates”^48^, relative to bregma. (S1FL Forelimb area of the primary sensory cortex). Bregma shown with a white square. (ii) Average voltage traces from the S1FL area in response to Paw stimulation from Control or ACh-lesioned mice. Vertical dashed line represents stimulus onset. (iii) Maximum depolarisation amplitude, Unpaired t-test, t _(117)_ = 2.706, *p* = 0.0078. (iv) Time of maximum depolarization, Mann–Whitney test, U = 1669, *p* = 0.5754. (v) Maximum hyperpolarisation amplitude, Unpaired t-test, t _(117)_ = 2.122, *p* = 0.0360. (**g**) (i) Through-skull cranial window with mapped areas according to “The Mouse Brain in Stereotaxic Coordinates”^48^, relative to bregma. (S1BF Barrel field of the sensory cortex). Bregma shown with a white square. (ii) Average voltage traces from the S1BF area in response to paw stimulation from Control or ACh-lesioned mice. Vertical dashed line indicates stimulation onset. (iii) Maximum depolarisation amplitude, Unpaired t-test, t _(117)_ = 0.4137, *p* = 0.6799. (iv) Time of maximum depolarization, Mann–Whitney test, U = 1411, *p* = 0.0528. (v) Maximum hyperpolarisation amplitude, Mann–Whitney test, U = 1624, *p* = 0.4430. All data are mean ± SEM. *p < 0.05, **p < 0.01, ***p < 0.001. NS non-significant. Scale bars 0.1% ΔR/R and 100 ms.

To account for the possibility that our results represented the consequence of systemic scopolamine blocking muscarinic ACh receptors elsewhere in the body, we depleted cortical ACh with a focal cortical injection of the specific cytotoxin mu p75 saporin (SAP) (Fig. 4a) a toxin that specifically destroys cholinergic fibres^58^. This ribosomal-inactivating cytotoxin is bound to an antibody directed against murine p75NRT uniquely expressed in the basal forebrain cholinergic neurons^59^. In this way we only lesioned ACh-releasing terminals/ fibres in the vicinity of the injection site 15 days after toxin administration (Fig. 4b). Although left and right forelimb use during simple exploration behaviour inside a glass cylinder (Fig. 4c) was normal in lesioned mice, they could not remain on the accelerating rotarod during several days of testing, unlike the control injected mice (Fig. 4d). These behaviour results suggest that our cortical lesion of cholinergic fibres only impaired more complex sensorimotor behaviours and motor learning.

Stimulation to the forepaw of ACh-lesioned mice evoked an initial strong depolarising response in S1FL that propagated caudally into the motor areas, as in controls, but was followed by a transient, prominent hyperpolarisation of all cortical areas peaking at around 180 ms after the stimulus (Fig. 4e and Supplementary Fig. 4, shows the pattern of depolarisation and subsequent hyperpolarisation in all cortical maps from individual mice). This different shape of the S1FL response pattern in ACh-lesioned mice is reminiscent of those from scopolamine treated mice, both significantly enhanced the amplitude of the initial depolarisation and the prominent slower, transient hyperpolarisation (Fig. 4f ii–v). We also observed a weaker, slower depolarisation of S1FL at 200–300 ms after stimulation (Fig. 4f ii) as also seen in scopolamine (Fig. 3e ii). Hyperpolarisation of S1BF in ACh-lesioned mice receiving forepaw stimulation remained (Fig. 4g ii–v). Side-by-side videos of responses to forepaw stimulation in mice 15 days after mu-p75SAP injection and control are found in Supplementary Video 3.

Our results provide compelling new evidence that in the awake animal, a muscarinic cholinergic mechanism controls the timing and pattern of spread of L2/3 cortical pyramidal neuron network activity during fast somatosensory cortical processing.

### Re-shaping of the forepaw sensory-evoked L2/3 pyramidal neuron responses by cholinergic fibre depletion alters the timing and direction of sensory adaptation

**Figure 5:**
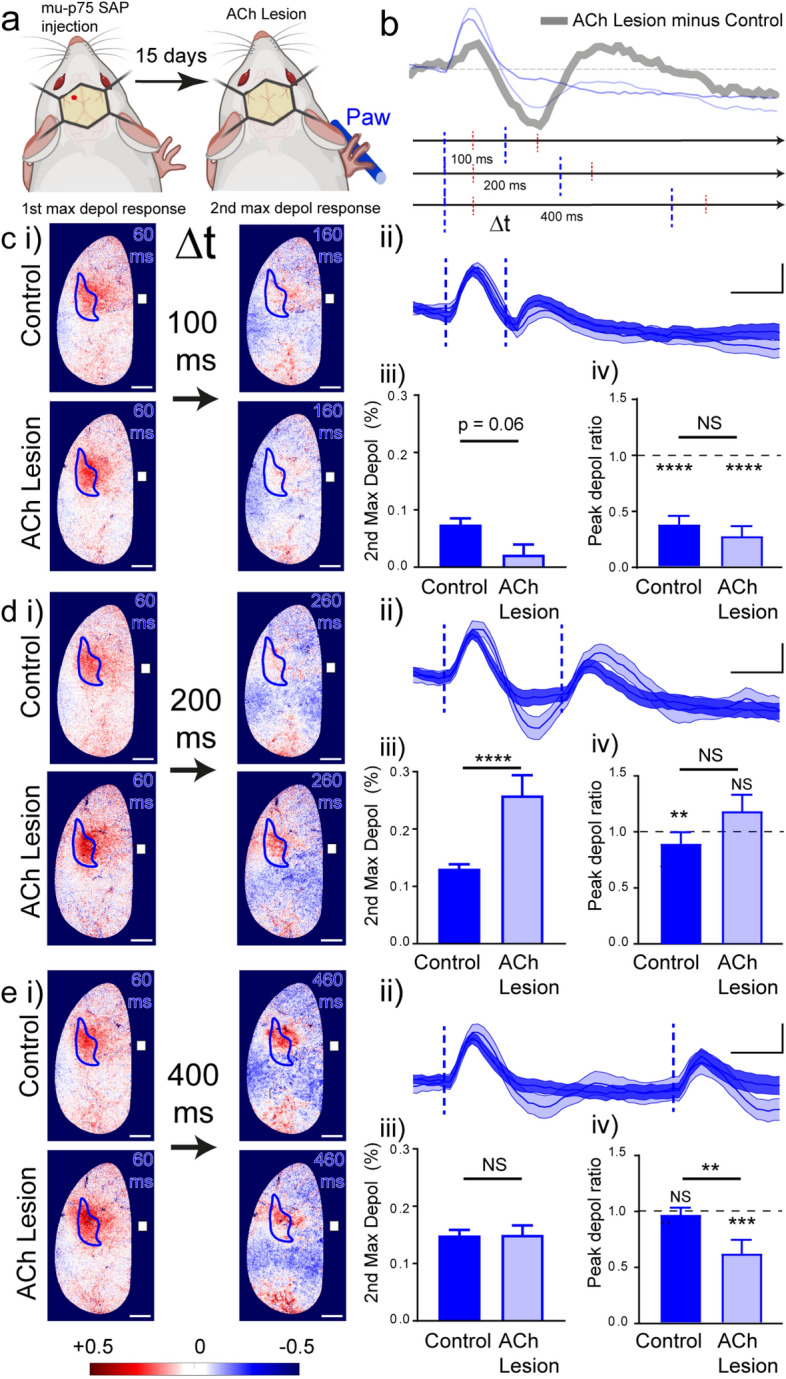
Lesion of cortical cholinergic fibres modifies sensory adaptation of tactile-evoked voltage responses in S1FL. (“Results”; “Discussion”). (**a**) Schematic methods. Mice received a unilateral cortical injection of mu-p75 SAP or IgG SAP Control into the left hemisphere, red dot indicates injection site. 15 days later mice were head-fixed and imaged during paw stimulation. Mouse images created with Biorender.com. (b) We assessed sensory adaptation by delivering two tactile stimulations to the Paw 100 ms, 200 ms or 400 ms apart (Δt) and compared the second response in forelimb area of the primary sensory cortex, S1FL with the first. Stimulation times are indicated by blue vertical dashed lines and times of peak depolarising responses in S1FL are indicated by red vertical dashed lines. Sensory-evoked voltage responses averaged from S1FL in control mice (blue) and ACh-lesioned mice (light blue). The thick grey trace is the result of subtraction of the responses from ACh-lesioned mice from control mice, and illustrates the times when the two responses are different, ie when the thick grey line is above or below the horizontal dashed line. Below, the stimulation protocol shows how the expected peak of the second response, second of red vertical lines, coincides with the times when the ACh-lesioned response was most different to control. (**c**) Peak depolarising responses in S1FL to second stimulation 100 ms after the first are smaller in both Control and ACh-lesioned mice. (i) Sensory-evoked voltage maps 60 ms after each of a pair of stimuli 100 ms apart. S1FL (blue) area. (ACh lesion n = 64 trials, 6 mice. Control n = 44 trials, 6 mice). Bregma shown with a white square. Scalebar 1 mm. (ii) Sensory-evoked voltage responses spatially averaged from S1FL. Vertical dashed lines represent stimulus onset. (iii) Amplitudes of the second depolarising peaks in mice with the ACh lesion and Control. Mann–Whitney test between groups, U = 1107, *p* = 0.0601. (iv) Peak depolarisation ratio (amplitude of the second depolarising peak/amplitude of the first depolarising peak). Wilcoxon Signed Rank Test, Theoretical value = 1, ACh lesion *p* < 0.0001, Control *p* < 0.0001. Mann–Whitney test between groups, U = 1195, *p* = 0.1848. (**d**) In contrast to D, the amplitude of the second sensory-evoked paw response (200 ms after the first response) in S1FL is larger in mice with the ACh lesion as compared to Control. (i) Sensory-evoked voltage maps 60 ms after each of a pair of stimuli 200 ms apart. S1FL (blue) area. Bregma shown with a white square. Scalebar 1 mm. (ACh lesion n = 33 trials, 6 mice, control n = 48, 6 mice). (ii) Sensory-evoked voltage responses spatially averaged from S1FL. Vertical dashed lines represent stimulus onset. (iii) Amplitudes of the second depolarising peaks in mice with the ACh lesion and control. Mann–Whitney test between groups, U = 451, *p* = 0.0005. (iv) Peak depolarisation ratio (amplitude of the second depolarisation peak/amplitude of the first peak). Wilcoxon Signed Rank Test, Theoretical value = 1, ACh lesion *p* = 0.5602, control *p* < 0.0074. Mann–Whitney test between groups, U = 640, *p* = 0.1460. (**e**) The ACh lesion reduces the sensory evoked responses from S1FL after a second paw stimulation 400 ms after the first stimulation. (i) Sensory-evoked voltage maps 60 ms after each of a pair of stimuli 400 ms apart. S1FL (blue) area. Bregma shown with a white square. Scalebar 1 mm. Depolarised pixels are shown in red and hyperpolarised pixels in blue. (ACh lesion n = 44 trials, 6 mice, control n = 41, 6 mice). (ii) Sensory-evoked voltage responses spatially averaged from S1FL. Vertical dashed lines represent stimulus onset. (iii) Amplitudes of the second depolarising peaks in mice with the ACh lesion and control. Mann–Whitney test between groups, U = 810, *p* = 0.4229. (iv) Peak depolarisation ratio (amplitude of the second depolarisation peak/amplitude of the first peak). Wilcoxon Signed Rank Test, Theoretical value = 1, ACh lesion *p* < 0.0001, control *p* = 0.3095. Mann–Whitney test between groups, U = 585, *p* = 0.0050. **c**(i)–**e**(i) Depolarised pixels red, + 0.5% ΔR/R and hyperpolarised pixels blue, − 0.5% ΔR/R. Data are mean ± SEM. Scale bars 0.1% ΔR/R and 100 ms.

We next sought to probe the significance of the re-shaped S1FL responses in ACh-lesioned mice, particularly the implications of the enhanced hyperpolarisation 110–200 ms after sensory stimulation (Fig. 5a,b). To do this we delivered a second stimulation to the forepaw at various times after the first stimulation, choosing times when the difference between the S1FL responses from control and ACh-lesioned animals was greatest (Fig. 5b). We predicted a reduction of the second response, particularly when the membrane was hyperpolarised. In line with this we observed a smaller depolarisation of S1FL to the second stimulation 100 ms after the first in control mice and an even smaller depolarisation in the ACh-lesioned mice (Fig. 5c i–iii). In both cases the peak depolarisation ratio was less than one (Fig. 5c iv) indicating that the second response was smaller than the first, and confirming efficient functional inhibition outlasting the first excitatory response (Fig. 1b i,d i). These results exemplify the well-established in vivo phenomenon of sensory adaptation, where a weaker response occurs following the second of two identical sensory stimulations^60^.

Surprisingly, in the ACh-lesioned mice, the depolarising response to a second stimulus with a 200 ms inter-stimulus interval was no longer smaller than the first (Fig. 5d i–iii) indicating little sensory adaptation in the absence of ACh at this interstimulus interval. Perhaps the reason for this is the underlying slow (200–300 ms), weak depolarisation of S1FL seen in response to a single forepaw stimulation in both scopolamine treated and ACh-lesioned mice (Figs. 3e ii, 4f ii).

In control mice, when we increased the interval between the pair of stimuli to 400 ms, the depolarising response to the second stimulus was almost as large as the first response (Fig. 5e i–iii). This was not the case for ACh-lesioned mice where the second depolarising response remained smaller than the first and was more dispersed across the cortex (Fig. 5e i–iii). Thus, sensory adaptation in ACh-lesioned mice lasted longer than in control (Fig. 5e iv), consistent with prolonged hyperpolarisation and possible shunting inhibition of the L2/3 pyramidal neuron outer dendrites^61^.

## Discussion

In the awake mouse, independent sensory tactile stimulation evoked distinct patterns of L2/3 cortical pyramidal neuron depolarisation and hyperpolarisation that propagated rapidly and widely across the cortex. A muscarinic cholinergic antagonist and cholinergic fibre depletion perturbed the timing and propagation of the sensory-evoked voltage responses, profoundly altering the timing of their adaptation, a universal phenomenon that underpins sensory discrimination^60,62^. These results provide fresh, new insights into how ACh influences sensory cortical processing to sustain sensory awareness and sensorimotor behaviours.

Brief, stimulation of the mouse forepaw, triggered a fast, depolarising L2/3 response in S1FL, whilst whisker stimulation rapidly depolarised S1BF neurons (Fig. 1b i,ii). The short (10 ms) time between the stimulus and the onset of L2/3 depolarisation likely represents fast sensory information transfer from the periphery to the cortex and then activation of the canonical cortical circuit^40,52,63^. After depolarisation, slower hyperpolarisations followed and spread widely across different cortical areas in response to both tactile stimuli, suggesting activation of independent long-range feed-forward L2/3 cortical inhibitory circuits. Combining whisker and forepaw stimulations (Fig. 2) confirmed the functional importance of this long-range inhibition for preserving the independence of whisker and forepaw cortical information streams^64–68^.

Sensory awareness in humans, and high-fidelity sensorimotor behaviours in animals and humans, require cortical ACh actions^12,69^. Tactile stimulation increases ACh levels in the cortex far more than other sensory stimuli^70^ and modifying ACh levels in the cortex in vivo alters L2/3 pyramidal neuron firing^32^. Yet, how ACh modifies integration of somatosensory L2/3 cortical pyramidal neuron activity in awake animals and its relevance for behaviour is not well understood. Therefore, we evoked tactile S1FL L2/3 pyramidal neuron membrane voltage responses in awake mice treated with scopolamine to acutely block muscarinic ACh receptors (Fig. 3) and after specific depletion of ACh fibres in the cortex (Fig. 4). Both treatments re-shaped the sensory-evoked L2/3 responses in S1FL in similar ways, indicating a predominantly muscarinic, cholinergic mechanism at work.

Scopolamine and ACh fibre depletion both increased the early depolarising response in S1FL. This early effect aligns with fast, phasic release of ACh into the cortex by sensory-evoked rapid increases in firing of basal forebrain cholinergic projection neurons^6,71,72^. Previous in vitro studies show that such rapidly released ACh excites cortical inhibitory interneurons including parvalbumin (PV) -positive, some vasoactive intestinal peptide (VIP) -positive and the classical-accommodating subtype of L1 interneurons in a muscarinic-dependent manner^20,73,74^, thus hyperpolarising L2/3 pyramidal neuron dendrites via feedforward inhibition. Whole-cell recordings from individual S1BF and visual cortex L2/3 pyramidal neurons in vivo also report increased amplitude of fast excitatory post-synaptic potentials by combined application of both muscarinic and nicotinic cholinergic antagonists^32,33^. Thus, we interpret our increased peak depolarising sensory-evoked S1FL response in the absence of ACh as removal of necessary muscarinic activation of fast, feed-forward inhibition. ACh fibre depletion also likely reduced actions of ACh at nicotinic receptors; notably, whisker and basal forebrain stimulation in vivo^74,75^ and stimulation that briefly elevates ACh in vitro^20,24^, excites VIP-positive and L1 interneurons.

Within the activated canonical cortical circuit, cholinergic-activated VIP-positive and Layer 1 (L1) inhibitory interneurons, whether by muscarinic or nicotinic activation, then inhibit PV-positive and SOM-positive inhibitory interneurons. Thus, ACh release disinhibits the cortex, indirectly exciting L2/3 pyramidal neurons^23,76^. Disinhibition is a universal mechanism for gain control and gating of excitatory cortical circuits^77^ often acting over long distance cortico-cortical circuits^78^ and is critical for many behaviours^79–81^. Here, removal of sensory-evoked muscarinic ACh-mediated disinhibition helps explain the observed pattern of slow, widespread hyperpolarisation of the L2/3 cortical pyramidal neuron network.

The similar patterns of sensory-evoked responses in S1FL in both scopolamine and ACh-lesioned mice indicate a dominant contribution from muscarinic modulation, although nicotinic actions may also contribute. Nevertheless, muscarinic cholinergic modulation dominates attentional rate modulation of visual-evoked frontal eye field broad spiking neurons of the awake macaque^82^. The arousal state of the mouse may also influence our results^16^. Previous studies suggest muscarinic cholinergic actions dominate when the cortex is in a low de-synchronised state^74^, as was likely in our awake, but quiet-trained mice. Likewise, nicotinic cholinergic activation of VIP-positive  interneurons^75^ disinhibits L2/3 pyramidal neurons in actively whisking mice when the cortex will be highly de-synchronised^74^. Further investigations are needed to assess the relative contributions of muscarinic and nicotinic actions for different sensory responses alongside meaningful measures of cortical state and arousal levels.

Furthermore, cholinergic actions in other layers of the cortex may also contribute to the re-shaping of sensory-evoked responses in L2/3^24,83^. Possibilities include cholinergic modulation of thalamo-cortical L4 to L2/3 connections^19,25^ and cortico-thalamic top-down modification of L2/3 sensory responses by cholinergic actions in L5^25^, or layer 6 (L6), thalamic projection neurons^84^. Pre-synaptic nicotinic receptor activation also improves activation of somatostatin (SST) -positive interneurons to enhance L2/3 pyramidal neuron feedback inhibition^85^, and activation of pre-synaptic muscarinic M2 auto-receptors can reduce ACh release^86^. Nicotinic ACh actions also lower the threshold for action potential initiation in thalamo-cortical axons, but the source of ACh and site of action are debated^87^. Non-neuronal astrocyte, glial and oligodendrocyte cholinergic actions may also influence sensory processing^73,87,88^.

Cortical cholinergic levels are known to drop during anaesthesia^4^ when cortical L2/3 pyramidal neuron firing synchrony also increases^33,89^. Previous studies in anesthetised mice show whisker-evoked initial depolarisation of S1BF cortical pyramidal neurons followed by a prominent, slow hyperpolarisation^47^, remarkably similar to the late, slow paw-evoked responses we see in awake ACh-lesioned and scopolamine-treated mice. More recently, a similar biphasic response of L2/3 pyramidal neurons to whisker stimulation is seen in quiet, non-whisking mice^75^. Furthermore, mecamylamine, a nicotinic ACh receptor antagonist, transforms the normally monophasic excitatory response in whisking mice to a biphasic, largely inhibitory response, in remarkably similar fashion to the transformation of our paw-evoked responses by muscarinic blockade or ACh-lesion. We can speculate that while sensory-evoked ACh promotes distinct nicotinic and/or muscarinic actions in different interneuron sub-types, the network design ensures that disinhibition by either or both mechanisms, offers sustained sensory-evoked L2/3 pyramidal neuron output.

The cumulative evidence in vivo therefore provides fresh new interpretations for how ACh modifies awake sensory processing. ACh first regulates the amplitude of the initial sensory-evoked depolarising L2/3 response (by fast feedforward inhibition) and then favours sustained activity of the L2/3 network (by timed disinhibition). We propose that by sustaining the reliable timing and broadcast of sensory-evoked L2/3 cortical activity patterns, ACh ensures the cortex is informed and prepared for upcoming sensory events.

To address how cholinergic modulation of L2/3 cortical activity might influence the processing of upcoming sensory events, we next focussed on the universal sensory phenomenon of sensory adaptation^90^. Adaptation is the reduced response to the second of two identical sensory stimuli and helps preserve sensory discrimination^90–94^. We observed a time window of adaptation, or reduction, of sensory-evoked S1FL depolarisations in control mice (Fig. 5) consistent with depression of excitatory thalamocortical synapses and recruitment of synaptic inhibition seen in vitro^90,95^. Without ACh, the amplitude of the second sensory-evoked response in S1FL (100 ms interval) decreased even further, indicating greater adaptation. Predictably, the greatest adaptation coincided with the peak of the slow S1FL hyperpolarisation, when inhibition is likely to be most powerful. Similarly during anaesthesia, when ACh levels drop^4^ adaptation of whisker behaviour also increases^96^. We suggest that ACh normally sustains a time window of L2/3 network activity to ensure proper response to a second sensory stimulus; such timing could be critical for working memory^57^. From a sensorimotor behavioural perspective, these actions may underlie the ability to discriminate a pair of closely-timed tactile stimuli. In humans, the shortest time interval necessary for a pair of tactile stimuli to be perceived as separate is called the somatosensory temporal discrimination threshold (STDT)^97,98^. STDT is significantly prolonged in early Alzheimer’s disease (AD) patients^99^, where cholinergic BF projection neurons degenerate first^17^. In AD, we predict the decline of cortical ACh unduly hyperpolarises the L2/3 broadcast network, leading to excessive adaptation, poor sensory discrimination and loss of sensory awareness.

Our findings may also relate to attention and its loss in early AD^100^. Similar to the excessive adaptation observed here, S1 pyramidal neuron firing in response to a distracter is reduced in rats with a cholinergic-depleted cortex, or when treated with muscarinic antagonists, indicative of reduced attention^101,102^. More directly, rats with a cholinergic depleted cortex exhibited reduced accuracy on a visual task that specifically requires sustained attention^103^.

Loss of muscarinic cholinergic signalling in AD may also underlie the observed reduction of the phenomenon of short latency afferent inhibition, SAI, in AD, and in healthy humans treated with muscarinic antagonists^104,105^. SAI is a somatosensory-motor phenomenon where prior, short latency (20–40 ms), somatosensory nerve stimulation (for example, of the hand) inhibits transcranial magnetic stimulation (TMS) of motor evoked potentials, MEPs, (for example, sent from the motor cortex to the muscles of the hand). SAI relies upon connections from S1 to M1, and the more excitable M1, the greater the likelihood of MEPs and therefore the weaker the SAI^104^. In mice treated with scopolamine, we observed a large amplitude, fast transient depolarisation of M1, entirely consistent with reduced SAI in humans.

In conclusion, fast voltage imaging, exclusively from the L2/3 cortical pyramidal neuron network, brings fresh new insights into how muscarinic cholinergic actions ensure accurate timing of awake, tactile sensory cortical processing. Our results provide an exciting springboard to examine how sensory-evoked ACh impacts other specific cortical populations, for example those carrying thalamo-cortical or cross-cortical sensory information, moving us another step closer to understanding the basis for sensory awareness in health and disease.

## Methods

### Animal ethics statement

All animal husbandry and ethical procedures in this study used protocols that were approved by the University of Otago Animal Ethics Committee (Animal Use Protocols: D07/16, D01/17, D35/17 and AUP-19-02) and were conducted in accordance with international ethical standards. We confirm that our work accords with the ARRIVE guidelines.

### Data and code availability

We developed a work routine using our own MATLAB 2018 scripts to process all the voltage imaging data and movement detection. These are available on request.

### Reagents, equipment suppliers

A comprehensive list of suppliers and sources of reagents and equipment are found in Table 1.

**Table 1:** Full list of reagents, equipment and suppliers.

	Source	Identifier
**Chemicals, peptides, and recombinant proteins**		
Sequence Cre Fwd 5′-CAC CCT GTT ACG TAT AGC CG-3′	Integrated DNA technologies	97513798
Sequence—Cre Rev 5′-GAG TCA TCC TIA GCG CCG TA-3′	Integrated DNA technologies	97513799
Sequence—tTA fwd 5′-CAA CCC GTA MC TCG CCC A.GA AG-3′	Integrated DNA technologies	97513800
Sequence—tTA Rev 5′-GGC CG.A AT.A AG.A AGG CTG GCT CT-3′	Integrated DNA technologies	97513801
Sequence—VSFP-B Fwd 5′-TCA AGG AGG CCG ACA MG AGA CC-3′	Integrated DNA technologies	97513802
Sequence—VSFP-B Rev 5′-ACA ACC AAC TGC CCC AAA CCA TC-3′	Integrated DNA technologies	97513803
Dream Taq Green PCR Master Mix(2x)	Thermo fisher	K1081
Trimethoprim (TMP)	Sigma-Aldrich	T7883
Tissue adhesive	3 M Vetbond	1469SB
DMSO	Sigma-Aldrich	D5879
Mu p75-SAP	Advanced Targeting Systems	IT-16
Rabbit IgG-SAP	Advanced Targeting Systems	IT-35
Isoflurane	Medscource NZ Ltd	VAPDRUGISO250
Carprieve	Norbrook NZ	200520
Ketamine	PhoenixPharm Distributors Ltd	9417
Domitor	Zoetis New Zealand Ltd	SKU 107334-9
Lopaine	Ethical Agents Veterinary Marketing Ltd, NZ	2010
Atropine	PhoenixPharm Distributors Ltd	9617
Temgesic	Indivior Pty Ltd, Australia	SKU IND00822 3060283
Low melting point agarose	Cleaver Scientific Ltd	A0701
Sodium chloride	Sigma-Aldrich	S7653
Potassium chloride	Sigma-Aldrich	P9333
Monosodium dihydrogen orthophosphate	Sigma-Aldrich	S8282
Sodium bicarbonate	Sigma-Aldrich	S6297
Calcium chloride	Fluka Analytical	21115
Magnesium chloride	Fluka Analytical	M2670
Monomer	Crown Dental & Medical Ltd	SMET0361
Polymer Clear	Crown Dental & Medical Ltd	SME0461
Catalyst V	Crown Dental & Medical Ltd	SME1281
Natural nail base coat	Naturliche Grundierung	0443011
Paraformaldehyde	Sigma-Aldrich	441244
Sucrose	neoFroxx	1104KG001
Sodium acetate	Sigma Aldrich	S7545
Acetylthiocholine iodide	Sigma Aldrich	A7571
Sodium citrate	Sigma Aldrich	S1804
Copper sulphate	Sigma Aldrich	209198
Potassium ferricyanide	Fulka	244023
Ammonium sulphide	Sigma Aldrich	A1952
Silver nitrate	Sigma Aldrich	S8157
Scopolamine hydrobromide	Tocris	1414
**Experimental models: organisms/strains**		
Triple Transgenic mice from crossing strains Rasgrf 2-2AdCre; B6;129S-Rasgrf2tm1(cre/folA)Hze/J Camk2a-ttA; B6.Cg-Tg(Camk2a-tTA)1Mmay/DboJ Ai78 Ai78(TITL-VSFPB1.2)-D or Ai78D	Jackson Labs JAX JAX JAX	022864 007004 023528
**Software and algorithms**		
Image J	Wayne Rasband NIH	1.51n
Prism	GraphPad	7
pClamp	Molecular Devices	10
MATLAB	MathWorks	2018a
MATLAB scripts bin_dir_satar.m make_mask.m Preprocessing.m lever_only.m seeds_ratio.m movement_detect.m area_ratio_data.m stim_analysis.m band_comb.m cortex_std.m		
Other		
Picospritzer	General Valve Co	52-302-900
TaskForcer licking unit	O’Hara & Co	OPR-LK
TaskForcer lever	O’Hara & Co	OPR-LV
TaskForcer Reward supply unit	O’Hara & Co	OPR-7300
Objective Nosepiece· for Two Objectives	SCTMEDIA	10450045
Handwheel for Motor Focus Drive	SCTMEDIA	10450298
CDAD Adaptor Set for Condensing Lenses	SCTMEDIA	CDAD
Olympus adaptor for I.ED	SCTMEDIA	AO-THT-OLY
acA1920-155 µm cameras	Basler AG	acA1920
Objective Planapo 1.0X M series	Leica	10450028
Plan Apo 0.63X'(WD67mm)	SCTMEDIA	10450027
Blue LED light	pE2, Cool LED	244-1400
Emission filter	Brainvision Inc	FF01-483/32-25
Dichroic mirror	Brainvision Inc	FF518-Di01
Dichroic mirror	Brainvision Inc	FF580-FDI
Emission filter	Brainvision Inc	FF01-542/27
Master-8-cp pulse timer	A.M.P.I. Instruments	4062
Low-Noise data acquisition System, Digidata 1550B	Axon Instruments, Molecular Devices	1550B
Rotarod	Ugo Basile	47650

A comprehensive list of sources of all supplies, equipment and software used.

### Experimental mice

Triple transgenic mice expressing Rasgrf 2-2AdCre; Camk2a-ttA; Ai78, 3 to 6 months old, from either sex were used in all our experiments. Triple transgenic mice (positive for dCre, tTA, and VSFP Butterfly 1.2 genes) express high levels of VSFP Butterfly 1.2 in pyramidal neurons of cortical L2/3 driven by the TRE (tetracycline response element) promotor^42,43^. The triple transgenic mice were selected based upon PCR-based genotyping from genomic DNA using the following primers^42,43^:

CRE 5′-ACCCTGTTACGTATAGCCG-3′ Forward.

CRE 5′-GAGTCATCCTTAGCGCCGTA-3′ Reverse.

tTA 5′-CAACCCGTAAACTCGCCCAGAAG-3′ Forward.

tTA 5′-GGCCGAATAAGAAGGCTGGCTCT-3′ Reverse.

Butterfly 5′-TCAAGGAGGCCGACAAAGAGACC-3′ Forward.

Butterfly 5′-ACAACCAACTGCCCCAAACCATC-3′ Reverse.

Non-triple transgenic littermates (VSFP Butterfly 1.2 non-expressers) were used for some experiments.

### Trimethoprim induction of gene expression in VSFP transgenic mice

The activity of dCre recombinase is low in the absence of trimethoprim (TMP) in VSFP Butterfly 1.2 mice; we induced full Cre activity by oral administration of TMP in raspberry jelly to activate VSFP Butterfly 1.2 expression^42^; 10 mg/ml TMP, in 1% DMSO/raspberry jelly mix; approximately 1 ml available each day.

### Scopolamine injections

Mice were injected intraperitoneally with scopolamine, muscarinic agonist (1 mg/kg) 30 min before the imaging session.

### Cholinergic lesion

We used mu p75 SAP (Advanced Targeting Systems) to remove the basal forebrain cholinergic fibres in the caudal forebrain area of the motor cortex^106^ and Rabbit IgG SAP (Advanced Targeting Systems) as a control molecule. mu p75 SAP or the control molecule was injected in M1 using the stereotaxic coordinates 0.3 mm anterior to bregma, 1.5 mm lateral to bregma and 0.3 mm depth from pia^48^. The toxin was injected in M1 to maximise cholinergic fibre loss in M1, M2 and S1, and to avoid a hole on the skull over S1, the main area of our interest for the optical imaging experiments.

### Surgical procedures

#### p75 SAP injection in the motor cortex to induce cholinergic lesion

Mice were anaesthetised with isoflurane (1–5%) and the analgesic non-steroid anti-inflammatory drug carprieve (5 mg/kg) given as pre-operative analgesia. After anaesthesia was established, the skin of the head was cut, and after gently removing the periosteum, one small hole drilled according to the coordinates above. mu p75 SAP or control molecule (1.7 mg/ml, 0.3 µl total volume, rate 0.075 µl/minute) was injected. The needle remained in place for a further 5 min before slow withdrawal over 2 min. The surgical incision was then closed with surgical sutures. Animals received carprieve (0.1 mg/kg) for two days after surgery.

#### Optical window preparation

Mice underwent surgical anaesthesia with ketamine (150 mg/kg) and domitor (1.5 mg/kg) or isoflurane, using 5% for anaesthesia induction with oxygen at 1 L/min, and 1–3% with the same oxygen flow for anaesthesia maintenance^107^. After anaesthesia was established, lopaine (4 mg/kg,) was applied subcutaneously above the incision area. Carprieve (5 mg/kg) and then atropine (0.05 mg/kg) were given subcutaneously before surgery. The skin of the head was cut and the skin edge carefully glued to the skull. After removing the periosteum the skull bone was thinned using a dental drill. A tiny piece of sterile black plastic was placed over bregma, as a reference point for registration of mouse brain cortical areas according to the atlas^48^. We secured the metal head-frame to the skull using dental cement. All the exposed bone was covered with a thin layer of dental cement and clear nail polish then applied.

Anaesthesia was reversed with anti-sedan (5 mg/kg). Animals received the postoperative opioid analgesic Temgesic (0.03 mg/kg, twice daily (with at least 6 h between administrations), for two days after the surgery and carprieve (0.1 mg/kg) once per day for four days after surgery.

#### Combined surgery of mu p75SAP injection and optical window preparation

Mice underwent surgery, as described above. After the skull was thinned using a dental drill, but before cement application, one small hole was drilled in the motor cortex, using the same stereotaxic coordinates. mu p75SAP or control molecule was injected in the stereotaxic coordinates. The hole was filled with 5% low melting point agarose dissolved in artificial cerebrospinal fluid; 126 mM Sodium chloride, 3 mM potassium chloride, 1 mM monosodium dihydrogen orthophosphate, 26 mM sodium bicarbonate, 2.4 mM calcium chloride and 1 mM magnesium chloride. All of the exposed bone and the agarose were covered with a thin layer of dental cement and then clear nail polish. The metal head-frame was secured to the skull using dental cement. Animals received postoperative analgesia as above.

### Behavioural experiments in freely moving mice

#### Cylinder test

We used the cylinder test to evaluate the ability of cholinergic lesioned and control mice to stand upright on their hind paws (rearings) and to test the symmetric use of their forelimbs. Mice were habituated to the test room the day before the experiment and then for 60 min on the day of testing, before the experimental session. Mice were placed into a cylinder glass container until they completed 20 rearings. The number of forelimb touches on the cylinder wall (with the left, right, or both forelimbs) and the time to complete 20 rearings were quantified. The asymmetry index (AI) was calculated as AI = (right forelimb contacts—1/2 both forelimbs contacts)/(right forelimb contacts + left forelimb contacts + both forelimbs contacts^108^.

#### Accelerating Rotarod

We tested cholinergic lesioned and control mice on the accelerating rotarod consisting of four trials per day for four consecutive days^109^. The rod, a mouse-specific apparatus accelerates from 5 to 40 rpm over 5 min and then remains at 40 rpm (revolutions per minute) for an additional 5 min for each trial. The latency time to fall from the rod was recorded up to a maximum of 10 min.

### Behavioural experiments in head-fixed mice

#### Water restriction and pre-training

Mice used for the imaging experiments were pre-trained to tolerate head fixation and paw and whisker stimulation. Mice implanted with the head frame were water restricted 15 days after surgery. Mice were head-fixed, and water given immediately after the mouse was removed from the head-fixing device twice per day. All mice undergoing water restriction were monitored twice daily for hydration, weight, ruffled fur, and normal behaviour. During water restriction we ensured that mouse body weight remained at greater than 90% of the initial weight.

#### Forepaw and whisker stimulation

Forepaw stimulation was delivered using a Task Force lever device. Mice were head-fixed for three days and habituated to hold the lever (pre-training) before the imaging session on day 4. A vibration (stimulation) of the lever was produced by rapidly unlocking (2 ms) and then locking the solenoids that control the lever. Lever vibrations were given with 30–60 s inter-trial intervals, 25 trials per day. Some mice received a double forepaw stimulation at 100, 200 and 400 ms interval. In control recordings the mouse was subjected to the same paradigm but the mouse was holding a lever that was not connected to the Task Force lever. Each mouse was head-fixed for a maximum of 25 min. Water was given immediately after each recording session.

The following week, mice were pre-trained over 3 days to tolerate whisker stimulation while head fixed, before imaging on day 4. Multiple whiskers were stimulated simultaneously using a brief air puff (10 psi, 20 ms) to the face delivered from a Picospritzer, via a 2 mm diameter metallic cannula placed approximately 1 cm in front of the right side of the face (Song, Piscopo et al. 2018); whiskers were not trimmed. Air puffs were given with 30–60 s inter-trial interval, 25 trials per day. Each mouse was head-fixed for a maximum of 25 min. Water was given immediately after each recording session, as in the paw stimulation experiments.

A subset of mice received forepaw stimulation followed by the air puff directed to the whiskers 60 ms later, or whisker stimulation and then paw stimulation 60 ms later.

### In vivo optical imaging

#### Widefield fluorescence imaging

Optical voltage imaging was performed as previously described^44^ with minor modifications. Image acquisition used a wide tandem lens epifluorescence macroscope equipped with a 1× objective and two synchronised acA1920-155 µm cameras in global shutter mode for dual-channel fluorescence imaging. One camera recorded the donor fluorescence, and the other camera recorded the acceptor fluorescence. Cameras were coupled to 0.63× lenses.

The recording optics included pass filters and beam splitters mCitrine (donor fluorophore) was excited with a blue LED light, 200–500 lx, passed through the emission filter (FF01-483/32-25). The excitation light was diverted onto the cortex via a dichroic mirror (FF518-Di01). A second dichroic mirror reflected the emitted fluorescence from mCitrine (FF580-FDI), passed through an emission filter (FF01-542/27) and collected by a camera. The emitted fluorescence from mKate2 (acceptor fluorophore) was transmitted by the second dichroic mirror, passed through an emission filter (BLP01-594R-50) and collected by the second camera. Camera acquisition times were synchronised with a Master-8-cp pulse timer.

During each sensory stimulation trial we acquired image sequences at 100 Hz, 1920 × 1200 pixel 12-bit resolution, 9.5 ms exposure time, 2 s long (200 frames) at 30–60 s intervals. 25 trials per day per mouse in whisker and paw stimulation experiments were recorded. In all our experiments, stimulation occurred 1000 ms after the excitation light was turned on (250 ms after the recording onset, allowing for acquisition of dark frames). Master-8-cp pulse timer synchronised camera activation, stimulus onset and excitation light and camera capture; image acquisition used a MATLAB 2018 script. Feed-back from the cameras (frame rate and frame grab time) was recorded at 10 kHz using a Low-Noise data acquisition System, Digidata 1550B controlled by pClamp10. The Master-8-cp activated the air-puff delivery, or lever vibration with confirmation of the stimulus time also recorded alongside the camera feed information by pClamp10, ensuring image frames aligned with the stimulation and behavioural events.

### Voltage signal analysis

#### Pre-processing of the images and voltage signal calculation

The initial voltage imaging signal pre-processing analysis used MATLAB scripts^44,47^. Donor and acceptor image sequences were first binned (factor 4) by averaging 16 pixels (4 × 4), resulting in 480 × 300 pixels images (pixel size 14.55 µm). The area outside the visible right hemisphere was masked out. Donor and acceptor fluorescence intensities were normalised on a pixel basis by the average of the pre-stimulus sequence (frame 30 to 120) after subtracting the camera offset (dark frames 1 to 25).

The pre-processing script then equalises the fluorescence intensity of donor and acceptor by rescaling them to each other based upon their relative amplitudes obtained from a fast Fourier transform of the data, where heartbeat rate is the dominant frequency^44^. The scripts perform the equalisation to every pixel of the donor and acceptor image sequences to equalise their amplitude. Voltage signals are then calculated as the acceptor to donor ratio (R) also on a pixel-by-pixel basis, resulting in ratiometric sequences for each pixel in each frame, as ΔR/R = (R − R0)/R0. R0 is the value of R averaged over 90 images preceding the stimulus (frames 30 to 120, 900 ms). Results are reported as a percent change in ratiometric activity (% ΔR/R = ([R − R0]/R0) * 100).

#### Removal of blood vessel signals

Prominent blood vessels appeared as dark vessels against the bright fluorescence of the expressed VSFP Butterfly 1.2. Using a MATLAB script, pixels corresponding to the vessels were removed by binarising a raw donor frame and excluding the dark, non-fluorescent zones of the images (superficial blood vessels and any minor imperfections on the cranial window) based on a threshold value. We used the same *inbinarise* threshold value for all animals based upon a previously empirically determined value from visual inspection of image sequences from 5 animals. Pixels that correspond to blood vessels were not averaged or used for any further calculations.

#### Cortical area registrations for each mouse with Franklin and Paxinos mouse brain atlas

We defined the cortical regions corresponding to S1 (forelimb, hindlimb, shoulder and barrel field areas), M1 and M2 based on “The Mouse Brain in Stereotaxic Coordinates”^48^. Then, we generated a colour coded cortical surface map with the atlas coordinates registered to bregma and the interhemispheric suture for each mouse. Our MATLAB script aligned the cortical map to the recorded brain images using bregma and the interhemispheric fissure as reference., and then generated spatially averaged voltage signals (ΔR/R) for all the pixels within each anatomically registered area of the cortex, frame by frame, for every imaging sequence trial. Voltage signals were then averaged across trials, stimulus conditions, and experimental groups.

#### Calculation of the amplitude and temporal parameters of the voltage signals for single stimulation experiments

The spatially averaged time-dependent voltage signals for each area of the cortex were analysed to detect the peak of the sensory-evoked responses. We identified and measured the peak amplitude of the depolarising response as the maximum local response (20–100 ms after the stimulus onset) relative to baseline (average of 10 frames, 100 ms before the stimulation onset). Trials with a maximum stimulation response amplitude less than two times the standard deviation of the baseline (10 frames before the stimulation) were excluded (Supplementary Fig. 1). The amplitude of the hyperpolarising response was measured as the minimum voltage detected (from the maximum depolarisation response to 300 ms after the stimulus onset) calculated relative to pre-stimulus baseline. We computed the temporal parameters of the sensory-evoked voltage responses as follows: peak (time to reach the maximum response) and decay (time to reach 50% of the maximum decay of the signal). All the above were automated within MATLAB.

#### Calculation of adaptation in double stimulation experiments

Using MATLAB, we calculated the peak amplitude of the depolarising response for each stimulation relative to the signal immediately preceding stimulation. We calculated the peak depolarisation ratio as the second evoked depolarisation amplitude divided by the first evoked depolarisation amplitude, as a measure of the relative adaptation^51^.

#### Spatial representation of the voltage signals across the cortex, voltage maps

We also generated voltage colour-coded maps by averaging all trials from all animals, frame by frame, on a pixel basis, and aligning all brains to their individual bregma, in MATLAB. We subtracted the membrane voltage (ΔR/R) matrix on a pixel basis from the baseline (average ΔR/R across ten frames, 100 ms before the stimulation, on pixel basis), ΔR = ΔR/R − ΔR/R baseline. ΔR for each pixel in the frame was then averaged with the corresponding pixels in other trials after aligning the images using bregma and the interhemispheric fissure as reference. The average membrane voltage (ΔR/R aligned) matrix was then plotted frame by frame, and the values scaled to the colour code in order to generate the colour-coded dynamic maps. We did not apply any additional spatial or temporal filtering.

#### Selection of trials based on mice behaviour movement and no movement

Mice were head-fixed as described in above during the recording sessions and behaviour was recorded using an acA1920-155um camera coupled to a TS1214-MP F1.4 Lens. The behavioural camera was synchronised with the cameras that recorded the donor and the acceptor fluorescence at 100 Hz by the Master-8-cp to ensure that fluorescence imaging and behaviour imaging aligned.

To detect mouse body movement on the trials, the absolute difference in the light intensity between each pixel in the frame captured when the stimulation was given (air puff or vibration) was compared with the corresponding pixels in the other frames. This was calculated ten frames before the stimulation and 60 frames after the stimulation, using MATLAB. To determine baseline absolute pixel intensity (noise without movement) we placed a stationary fabric mouse in the head holder (size and shape to mimic a real mouse) and the absolute difference in the light intensity determined between the frames calculated as described above. This method allowed to classify that a pixel intensity changed when the difference compared to the stimulation frame was higher than the change between baseline and the stationary fabric mouse. Supplementary Fig. 2A shows the pixels that change in four representative frames compared to the stimulation frame, for a living mouse that moved.

The percentage of pixels per frame with intensity differences (compared to the stimulation frame) that were higher than the baseline intensity changes was calculated. Empirically, 0.5% of changed pixels separated significant visible movement from little to no visible movement, by visually examining 75 trials from 3 mice independently and classifying each frame (200 frames) of the behavioural video into movement or quiet (Supplementary Fig. 2). The visual inspections were compared with the calculated values of the percentage of pixels that change per frame arriving at a value of 0.5% when body movement did not occur. Any movement under this empirical value of 0.5% represents breathing or other minor movements of a quiet mouse.

### Anatomical methods

#### Perfusion and fixed tissue preparation

At the end of the experiments, animals were euthanised using ketamine (150 mg/kg) and domitor (2 mg/kg) and perfused with 4% paraformaldehyde, ~ 5 ml/minutes flow. The brain was removed, post-fixed in 4% paraformaldehyde overnight and cryoprotected in 30% sucrose in deionised water for at least another 48 h.

30 µm thick sections were cut in the coronal plane on a cooled (dry ice) sledge microtome (Leica SM2400, Germany), and sequential sets of sections collected.

#### Acetylcholinesterase staining

We visualised ACh fibres by their expression of acetylcholinesterase detected using histochemistry performed with silver nitrate intensification^110^. Brain slices were incubated in sodium acetate buffered (0.1 M; pH 6, Sigma Aldrich) acetylthiocholine iodide (0.05%, Sigma Aldrich), sodium citrate (0.1 M, Sigma Aldrich), copper sulphate (0.03 M, Sigma Aldrich), and potassium ferricyanide solution (5 mM, Fulka), ammonium sulphide (1%, Sigma Aldrich) and then silver nitrate (1%, Sigma Aldrich). Then, slices were mounted on glass slides, briefly air-dried, and coverslipped with slide mounting medium for histology (Sigma-Aldrich).

#### Cholinergic fibre quantification

We imaged acetylcholinesterase stained brain slices (Olympus BX-51 microscope, 1280 × 1024 pixels, and 0.32 μm/pixel) keeping microscope and acquisition parameters consistent.

The analysis of acetylcholinesterase positive fibres used images taken in M1, M2 and S1 using the “The Mouse Brain in Stereotaxic Coordinates”^48^ to localise the cortical areas. Ten cortical sections were analysed, with rostral-caudal intervals of 150 μm (every 5th slice), in each animal, from ~ 0.10 mm posterior to bregma to ~ 1.42 mm anterior to bregma^48^.

Cholinergic fibre quantification for acetylcholinesterase was performed as previously described^111^, setting a grey-scale threshold to measure the area of cholinesterase positive fibres (the area covered by positive cholinergic fibres) using Image J software^112^. Cortical projections from the basal forebrain occur independently within each hemisphere, so the contralateral, non-lesioned side of the brain served as a within-subject control for each mouse. Thus, cholinergic fibre reduction on the lesioned side was calculated as a percentage difference between the area of fibres detected on the lesioned and non-lesioned sides of the same brain^113^.

### Experimental design and statistics

Within Subject Factors—Whisker and Forelimb Tactile Evoked Sensory Responses from multiple brain regions under control and cross modal stimulation; Adaptation, responses from S1FL at different stimulation time intervals.

Between Subject Factors—Cholinergic Lesion versus Vehicle Controls, Muscarinic Antagonist versus non-treated Controls, GEVI-expressing versus non-transgenic, non-expressers. Animal numbers, trial numbers and sex are shown in Supplementary Figs. 3 and 4.

We determined the normal distribution for all datasets using a D'Agostino & Pearson normality test. Data that did not pass the normality test or where the N was too small used subsequent non-parametric statistic tests. Group data were averaged and reported as means ± SEM. For two-group comparisons, normal data was analysed using a t-test and non-parametric data with a Mann–Whitney test. One-way ANOVA and Tukey’s post hoc test were used for pair-wise statistical comparisons between three or more groups for parametric data, and Kruskal–Wallis test and Dunn's multiple comparisons for non-parametric data. For comparisons of multiple parameters between groups, a two-way ANOVA followed by Tukey's multiple comparisons test was used. Wilcoxon Signed-Rank Test compared a group with a theoretical value. All live imaging data were processed using MATLAB (R2018a) and Microsoft Excel (Professional Plus). Statistical analyses used Prism (GraphPad Prism7 Inc). Data was reported as significant if *p* < 0.05.

## Supplementary Information

Supplementary Information.

Supplementary Video 1.

Supplementary Video 2.

Supplementary Video 3.

Supplementary Table 1.
