# Supplementary material for: Cholinergic modulation of sensory processing in awake mouse cortex
Source: Sci Rep. 2021 Sep 1;11:17525. doi: 10.1038/s41598-021-96696-8 (PMC8410938; doi:10.1038/s41598-021-96696-8)
Supplement: Supplementary file 1 — Supplementary Information. [file 41598_2021_96696_MOESM1_ESM.pdf]

Supplementary Figures and Figure Legends

Supplementary Figure 1 Stepwise trial selection based on no detectable movement / movement and signal amplitude above noise

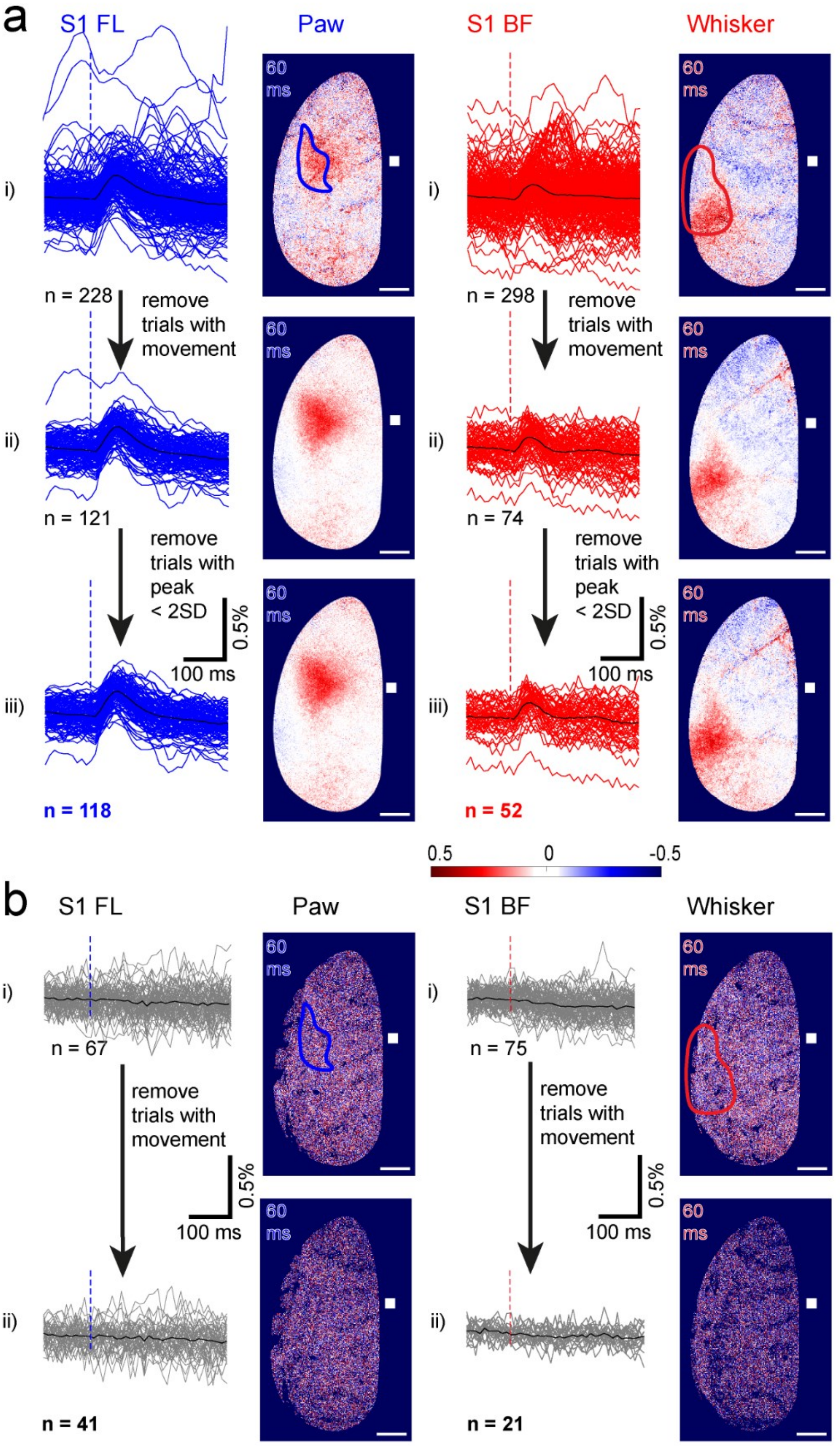

Legend to Supplementary Figure 1

(a)

i) Sensory-evoked voltage response single traces from the forelimb area S1FL and the barrel field S1BF of the sensory cortex, and maps (pixel by pixel changes in VSFP Butterfly 1.2 donor and acceptor and area average responses ΔR/R, ratio % change vs baseline) after paw and whisker stimulation respectively. Average ratio is shown in black. The vertical dashed line indicates the time of stimulation onset.

ii) Trials, where mice moved above the predefined movement threshold (see Suppl Fig 1B), were initially excluded from the analysis.

iii) A second refinement removed all trials with a peak amplitude after stimulation of <2SD of the baseline (SD calculated from 10 frames (100 ms) immediately before the stimulation), leaving 118 paw stimulation trials and 52 whisker stimulation trials. These trials then formed the basis for all further analysis.

(b) Non-triple, without VSFP Butterfly 1.2 expression, littermate mice showed no responses after whisker or paw stimulation. Sensory-evoked voltage maps and ratiometric recordings after paw and whisker stimulation (arrowhead) in the forelimb area and the barrel field of the sensory cortex, grey traces are single trials, average is in black. Data are mean ± SEM.

i) Paw and whisker stimulation: 3 mice in each group performed 25 trials giving 75 total, in 8 of the paw stimulation trials the mouse was not holding the lever leaving 67 trials for analysis.

ii) Trials where the mouse moved after paw or whisker stimulation were excluded, leaving n = 41 and n = 21 trials respectively.

Supplementary Figure 2 Widespread optical signals associated with mouse body movement in response to sensory stimulation.

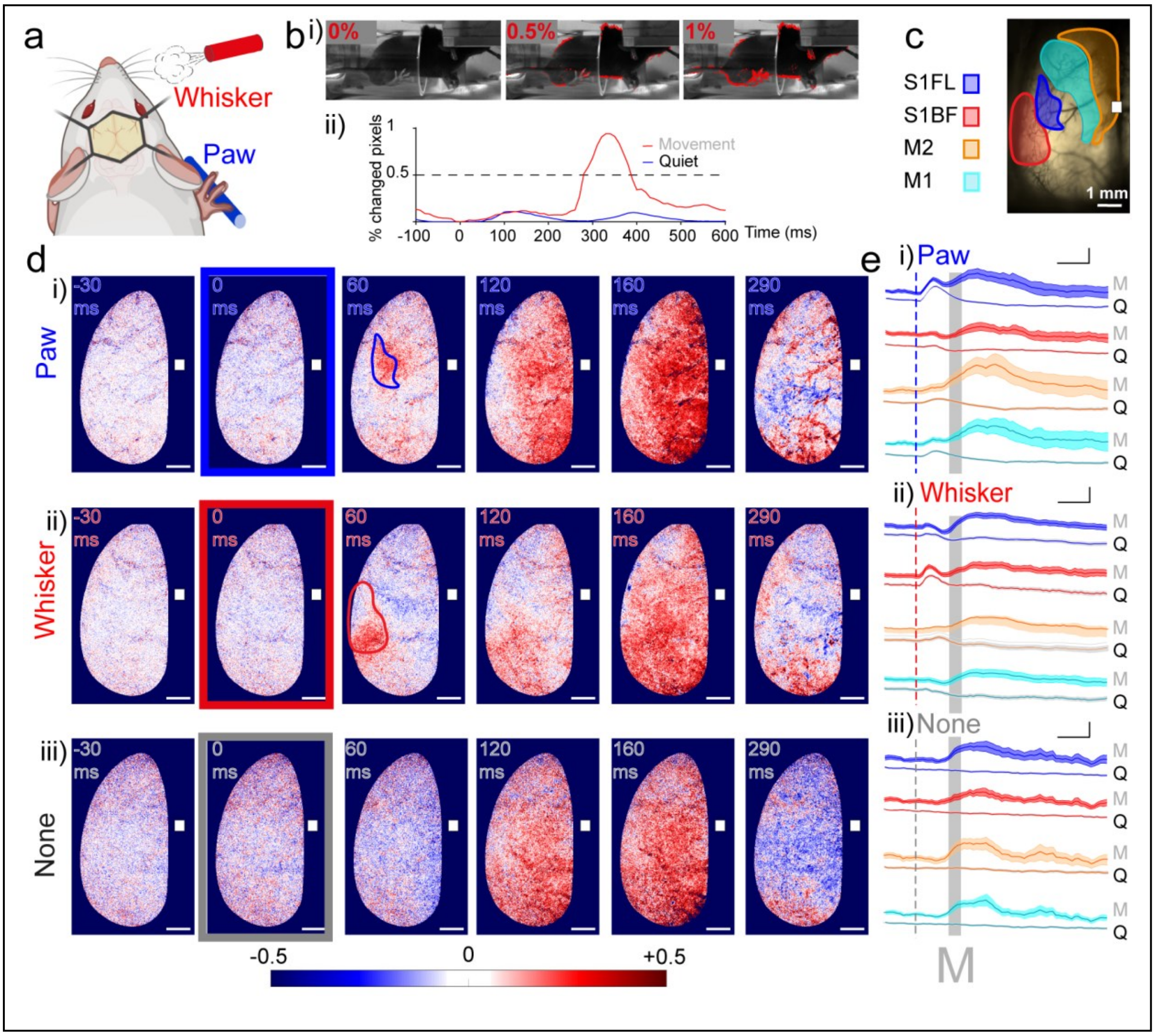

Legend to Supplementary Figure 2

(a) Diagram of awake mice expressing VSFP Butterfly 1.2 in layer 2/3 cortical pyramidal neurons, with thinned-skull cranial window responds to paw, and whisker stimulation.

(b) Mouse movement detection using a pixel-based threshold method. Mouse image created with Biorender.com.

Top: cropped frames of the head-fixed mouse (for ease of display) with red pixels marked where pixel intensity changed above the baseline (no mouse), frame by frame. 0.5% used to separate significant visible movement, labelled "Movement", from little to no visible

movement, labelled "Quiet"; note the higher number of red pixels at 1% where the mouse clearly moves its right hind paw.

Bottom: Frame by frame the percentage of pixels that change throughout an example trial. Excluded "Movement" trials showed pixel changes greater than 0.5% threshold (red trace). Note the small, slow increase and decrease in the number of pixels on a time scale consistent with breathing in the included, "Quiet" trial (blue trace).

(c) Through-skull cranial window with mapped areas according to "The Mouse Brain in Stereotaxic Coordinates" (Franklin and Paxinos, 2008), relative to bregma. (M1, Primary motor cortex; M2, secondary motor cortex; S1FL Forelimb area of the primary sensory cortex, S1BF Barrel field of the primary sensory cortex). Bregma shown with a white square.

(d) Sensory-evoked voltage maps (pixel by pixel changes in ΔR/R, ratio change vs baseline) in trials where mice moved 100 – 140 ms after a single forepaw stimulation (i), air puff to the whiskers (ii) and control no stimulation, none, trials (iii) at selected times before (-) and after stimulation; coloured frame is the stimulation frame. Scale bar is 1 mm. Depolarised pixels red, +0.5% ΔR/R and hyperpolarised pixels blue, -0.5% ΔR/R. Movement evoked a widespread depolarization throughout the dorsal cortex.

(e) Sensory-evoked voltage responses (spatial area average of ratio values for all pixels within area, ΔR/R, ratio % change vs baseline) from S1FL (blue), SIBF (red), M2 (orange) and M1 (cyan) in response to forepaw stimulation (i), whisker stimulation (ii) and control trials with no stimulation (iii). Trials with "Movement" (grey, M) trials with no movement, "Quiet" (black, Q). Same trials as in D. Vertical dashed line indicates stimulation onset.

Data are mean ± SEM. Scalebar is 0.2% ΔR/R and 100 ms. Movement occurred 100 -140 ms after paw stimulation (vertical grey bar, M)

No movement: paw stimulation n = 118 trials, 12 mice. Whisker stimulation n = 52 trials, 10 mice. No stimulation n = 152 trials, 12 mice

Movement: paw stimulation n = 19 trials, 7 mice. Whisker stimulation n = 15 trials, 9 mice.

No stimulation n = 15 trials, 9 mice.

Supplementary Figure 3 Distinct membrane voltage responses from the forelimb area and the barrel field following forepaw and whisker stimulation in all individual mice.

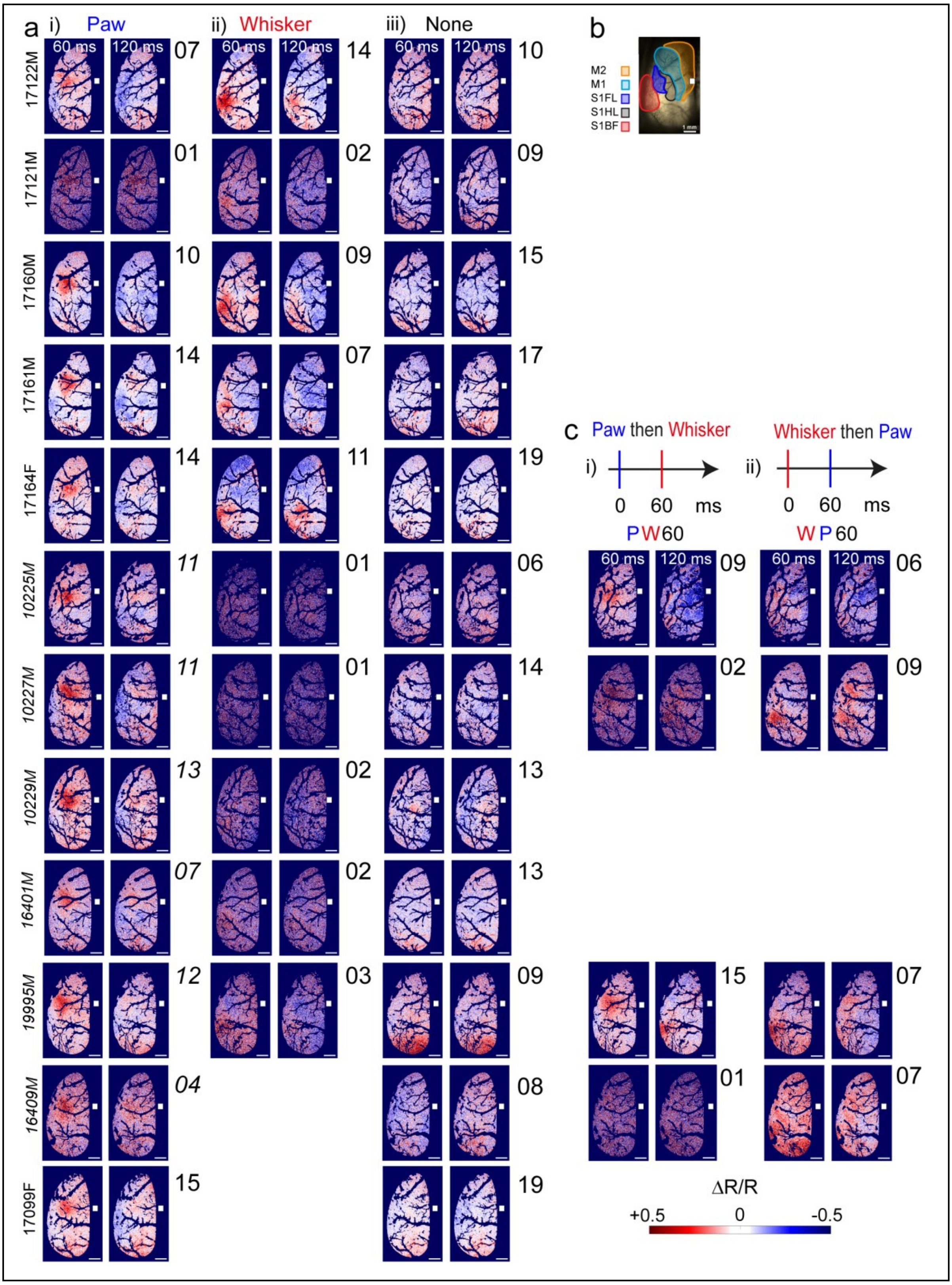

Legend to Supplementary Figure 3

(a) Sensory-evoked voltage maps in response to forepaw stimulation (i), air blown to the whiskers (ii) and control no stimulation trials (iii) 60 and 120 ms after stimulation. Forepaw stimulation evoked a depolarization in the S1FL (outlined in blue) 60 ms after the stimulation and a hyperpolarization of the S1BF (outlined in red) 120 ms after the stimulation. Whisker stimulation evoked a depolarization in the S1BF 60 ms after the stimulation and a hyperpolarization in the S1FL 120 ms after the stimulation.

(b) Through-skull cranial window with mapped areas according to "The Mouse Brain in Stereotaxic Coordinates" (Franklin and Paxinos, 2008), relative to bregma. (M1, Primary motor cortex; M2, secondary motor cortex; S1HL, Hindlimb area of the primary sensory cortex; S1FL, Forelimb area of the primary sensory cortex; S1BF, Barrel field of the primary sensory cortex). Bregma indicated with a white square.

(c) Sensory-evoked voltage maps in response to multimodal sensory stimulation in individual mice. "paw then whisker (P then W)" experiments (i) the forepaw was first stimulated and then 60 ms later air puff was delivered to the mouse whisker. "whisker then paw (W then P)" experiments (ii) whisker was fist stimulated and then forepaw was stimulated 60 ms later.

Scale bar = 1 mm. Depolarised pixels red, +0.5% $\Delta R/R$ and hyperpolarised pixels blue, -0.5% $\Delta R/R$. Vertical numbers are individual animal id numbers and horizontal numbers are the number of response trials used to generate each individual map. Vertical italicized ids are mice subsequently treated with scopolamine see Supplementary Figure 4.

Supplementary Figure 4 Acute scopolamine and chronic cholinergic lesion re-shapes the sensory-evoked voltage response maps 60 and 180 ms after forepaw stimulation in all individual mice.

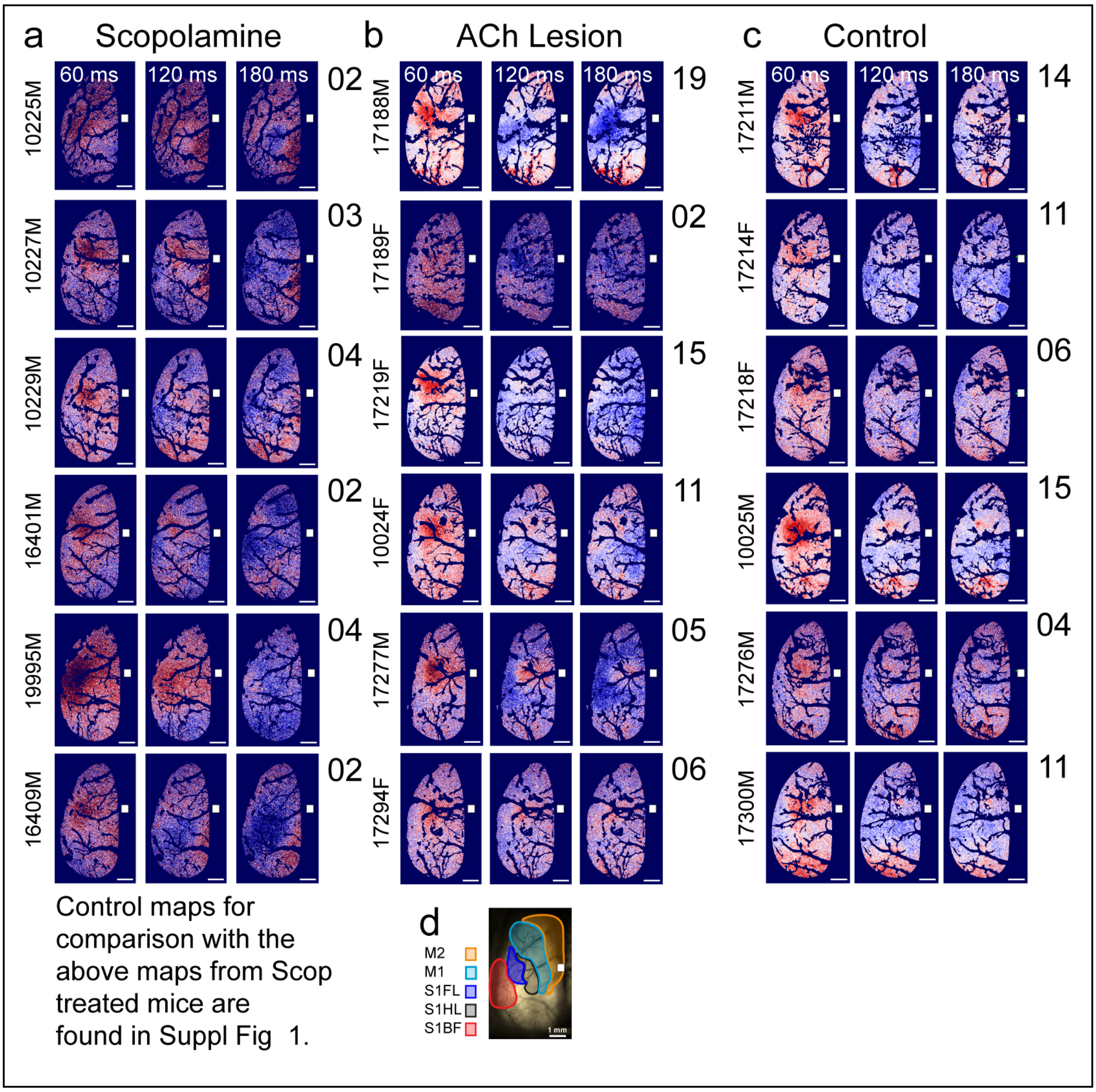

Legend to Supplementary Figure 4

(a) Sensory-evoked voltage maps from mice 30 minutes after intraperitoneal injection with scopolamine.

(b) Sensory-evoked voltage maps from mice 15 days after a cholinergic lesion generated by injection of p75 saporin in the sensorimotor cortex

(c) Sensory-evoked voltage maps from mice 15 days after injection with non-targeted saporin in the sensorimotor cortex

(d) Through-skull cranial window with mapped areas according to "The Mouse Brain in Stereotaxic Coordinates" (Franklin and Paxinos, 2008), relative to bregma. (M1, Primary motor cortex; M2, secondary motor cortex; S1HL, Hindlimb area of the primary sensory cortex; S1FL, Forelimb area of the primary sensory cortex; S1BF, Barrel field of the primary sensory cortex). Bregma indicated with a white square.

Scale bar = 1 mm. Depolarised pixels red, +0.5% $\Delta R/R$ and hyperpolarised pixels blue, -0.5% $\Delta R/R$. Vertical numbers are individual animal identity numbers and horizontal numbers are the number of response trials used to generate each individual map.

Supplementary Videos

Supplementary Video 1 Paw Whisker responses side by side

Supplementary Video 2 Scopolamine and control responses side by side – paw

Supplementary Video 3 ACh-lesion and control responses side by side – paw
