# Supplementary material for: Cholinergic modulation of sensory processing in awake mouse cortex
Source: Sci Rep. 2021 Sep 1;11:17525. doi: 10.1038/s41598-021-96696-8 (PMC8410938; doi:10.1038/s41598-021-96696-8)
Supplement: Supplementary file 5 — Supplementary Table 1. [file 41598_2021_96696_MOESM5_ESM.pdf]

Supplementary Table 1. Time to maximum depolarization of the spatially averaged cortical areas during whisker and paw stimulation.

Stim	Cortical area										
	M1		M2		S1BF		S1FL		S1HL		Friedman test ANOVA
	Time to peak (ms)	Dunn's Multiple Comparison	Time to peak (ms)	Dunn's Multiple Comparison	Time to peak (ms)	Dunn's Multiple Comparison	Time to peak (ms)	Dunn's Multiple Comparison	Time to peak (ms)	Dunn's Multiple Comparison	
Whisker	[$#]45.58 ± 2.98	$p > 0.99$<br>$p = 0.55$	[§]65.19 ± 3.08	$p = 0.0002$<br>$p = 0.06$	50.58 ± 1.95	$p = 0.43$	[#]47.31 ± 2.76	$p = 0.43$	[#]47.50 ± 2.85	$p > 0.99$<br>$p > 0.99$	$Q_{(4)} = 28.00$, $p < 0.001$
Paw	[$]61.27 ± 1.67	$p = 0.87$<br>$p < 0.0001$	[§]66.36 ± 2.28	$p = 0.011$<br>$p < 0.0001$	[#]46.10 ± 1.85	$p < 0.0001$	59.83 ± 1.13	$p < 0.0001$	61.27 ± 1.42	$p > 0.99$<br>$p < 0.0001$	$Q_{(4)} = 88.34$, $p < 0.001$

Legend to Table 1

Friedman nonparametric repeated measures ANOVA, and Dunn's multiple comparisons test, comparison with the S1FL on the top of the cell (in blue) and comparison with the S1BF on the bottom (in red)
(M1, Primary motor cortex; M2, secondary motor cortex; S1HL, Hindlimb area of the primary sensory cortex; S1FL, Forelimb area of the primary; Sensory cortex; S1BF, Barrel field of the primary sensory cortex.

Note that both Whisker and Paw stimulation evoke similar depolarisation times to peak of area M2[§] ($p = 0.67$, Mann-Whitney), but not M1[$] ($p < 0.0001$, Mann-Whitney), compare also Fig. 1 d (i) and (ii), cyan and orange traces.

The apparently fast times to peak of S1BF after Paw stimulation and S1FL, S1HL and M1 after Whisker stimulation[#], are likely a consequence of curtailed depolarisation by the onset of strong hyperpolarisation in these areas, compare also Fig. 1 d (i) and (ii) red, blue, grey and cyan traces.
